# A quantum-chemical study of synthesis and stability of glycine on olivine surface

**DOI:** 10.1007/s00894-026-06745-0

**Published:** 2026-05-07

**Authors:** Abu Asaduzzaman

**Affiliations:** School of Science, Engineering and Technology, Pennsylvania State University Harrisburg, Middletown, PA 17057 USA

**Keywords:** Astrochemistry, Amino acid, Surface chemistry, Quantum chemistry

## Abstract

**Context:**

Using glycine as a model system, this study examines the surface assisted reaction between methylamine and formic acid on an olivine (010) surface. A comparative analysis of co-adsorbed, mixed phase, and gas phase pathways of methyl amine and formic acid to form glycine shows that a surface assisted pathway exists that connects adsorbed formic acid and methylamine to a surface stabilized glycine configuration with significantly reduced reaction energetics relative to gas phase, suggesting a plausible role for silicate minerals in facilitating complex organic synthesis under astro-physically relevant conditions.

**Methods:**

First-principles density functional theory calculations were performed using the Vienna Ab initio Simulation Package (VASP) with the Perdew–Burke–Ernzerhof (PBE) exchange correlation functional and the projector augmented wave (PAW) method. Minimum energy reaction pathways and activation barriers were determined using the nudged elastic band (NEB) method, with all energies referenced to clearly defined initial states. Short time thermal stability of surface-bound glycine was assessed using ab initio molecular dynamics (AIMD) simulations at 1200 K to probe high temperature persistence on picosecond timescales.

**Supplementary Information:**

The online version contains supplementary material available at 10.1007/s00894-026-06745-0.

## Introduction

The availability, synthesis, and spatial distribution of organic compounds within the protoplanetary disk have long been central themes in astrochemical research. Organic matter in the early solar system is thought to originate primarily from interplanetary dust particles (IDPs), meteorites, comets, and asteroids [1–4]. Together, these reservoirs provide critical insights into the chemical complexity that existed prior to planetary formation.

A growing body of experimental and observational studies [5–18] has demonstrated that organic material is often intimately associated with inorganic matrices in primitive solar system matter. For example, core–shell nanoglobules consisting of organic-rich cores encapsulated within inorganic shells have been identified in carbonaceous chondrites, as reported by Garvie et al. [7] and Nakamura et al. [19]. In addition, in situ analyses have revealed the presence of organic films both between mineral grains and on mineral surfaces [8–11]. Together, these observations indicate that organic compounds are not merely superficial contaminants but are structurally integrated into primitive solar system materials.

Further chemical analysis of relatively unaltered IDPs indicates that primitive solar system dust can harbor substantial quantities of organic matter [12–14]. These organics are often incorporated into sub-micron-sized grains, highlighting the potential for widespread distribution of complex molecules even at the earliest stages of planetary accretion.

Remote spectroscopic observations have revealed a diverse inventory of volatile organic molecules in comets. Rotational and vibrational spectroscopy has detected numerous species in cometary comae, including those from long period and Jupiter family comets [5, 15, 16]. Specifically, the Rosetta mission identified a rich suite of organic compounds on the surface of comet 67P/Churyumov-Gerasimenko using onboard mass spectrometry, while the Stardust mission returned samples from comet 81P/Wild 2 containing glycine [18]. These findings underscore the role of comets as reservoirs and potential delivery vehicles of prebiotic organics to early Earth. In 2024, Manna and Pal [20] reported the detection of methylenimine (CH₂NH), a key precursor to glycine, in the hot molecular core G10.47 + 0.03, reinforcing the plausibility of amino acid formation in interstellar environments.

Despite this wealth of data, the mechanisms governing the formation of organic compounds and their association with inorganic grains remain incompletely understood. Several hypotheses have been proposed to explain these processes. One prominent pathway involves Fischer–Tropsch–type (FTT) reactions occurring on catalytically active mineral surfaces within the protoplanetary disk [21]. Another mechanism involves aqueous alteration on parent bodies, where fluid–rock interactions can promote the synthesis and transformation of more complex organic compounds [22].

Moreover, ultraviolet (UV) irradiation of icy grains in the cold outer regions of the protoplanetary disk (with temperatures below 30 K) has been shown to drive the formation of both simple and complex organic molecules [23]. Laboratory irradiation experiments and complementary theoretical models support the view that such photochemical processing in the solar nebula can generate a diverse inventory of prebiotic compounds. More recently, Jarois et al. [24] proposed a novel *aza-Cannizzaro* mechanism for glycine formation from glyoxylic acid in ammonium-rich aqueous environments, offering new insight into the incorporation of nitrogen into prebiotic organic chemistry.

The investigation of amino acid synthesis and its relevance to the origin of life can be traced back to the pioneering Urey–Miller experiments [25, 26], which demonstrated that organic compounds could be produced under simulated prebiotic conditions. Since then, numerous experimental studies [27] have examined the plausibility of abiotic amino acid formation under a range of early Earth scenarios. In most of these investigations, small molecules such as CO, CO_2_, NH_3_, CH_4_, H_2_O, and HCN were used as reactants and energized by external sources including electric discharges, ultraviolet radiation, or thermal input to mimic natural processes such as lightning, solar irradiation, or volcanic activity. In parallel, a number of theoretical studies [28–31] have explored the thermodynamic and kinetic feasibility of amino acid synthesis under planetary formation conditions, similarly focusing on reactions involving small molecules or reactive radical species. More recently, however, Ishikawa and Sato [32] reported the successful synthesis of glycine and alanine from amines and formic acids under ultrahigh-pressure conditions generated by laser-driven shock waves. This result highlights the potential for direct amino acid formation from amines and carboxylic acids, thereby broadening the range of plausible prebiotic synthetic pathways.

Mineral surfaces, particularly those of silicates like olivine, are increasingly recognized as potential catalysts for prebiotic reactions. Specifically, Hill and Nuth [33] demonstrated that simple gas phase species detected in the inner solar nebula such as CN, HCN, C₂H₂, OH, CO, and CO₂ [34, 35] can undergo surface mediated reactions on mineral grains. These reactions can produce a diverse set of complex organics, including long chain aliphatic, aromatic hydrocarbons, amines, carboxylic acids, amides, and imines, all of which are relevant to prebiotic chemistry.

To deepen understanding of the interactions between inorganic grains and organic molecules, a first-principles DFT study was recently conducted to investigate the adsorption behavior of simple organic species on representative meteoritic surfaces [36]. Specifically, the interactions of methane, ethylene, benzene, methyl cyanide, methylamine, and formic acid with several mineral surfaces commonly found in meteorites including olivine, water ice, magnesite, and spinel were systematically examined. These results demonstrated that organic molecules containing electronegative functional groups, particularly those incorporating nitrogen or oxygen atoms, tend to form chemical bonds with undercoordinated surface atoms of the inorganic substrates. Such bonding is driven by a combination of electrostatic and covalent interactions between surface cations and the electronegative atoms within the organic molecules. Analysis of the adsorption energies showed that these interactions are sufficiently strong to suggest that organic compounds could have been delivered to the early Earth during planetary accretion, potentially surviving the extreme conditions associated with impact and thermal processing.

Building on these findings, the current study aims to explore whether strongly adsorbed organic molecules can undergo surface catalyzed reactions to form more complex prebiotic compounds. Using DFT, I investigated the formation of glycine, the simplest amino acid, through a surface-mediated reaction between adsorbed methylamine and formic acid. To assess the catalytic role of the mineral surface, I also simulate the gas phase formation pathway of glycine, enabling a plausible comparison between surface assisted and non surface assisted mechanisms. Furthermore, I examine the temperature dependent stability of glycine on the mineral surface to mimic the thermal conditions of the protoplanetary disk environment. For this purpose, olivine, a silicate mineral and a major component of Earth’s crust and many meteorites, have been selected as a model surface due to its geochemical relevance and catalytic potential.

## Computational methods

Quantum-chemical calculations were carried out using the Vienna Ab initio Simulation Package (VASP) [37, 38]. Structural optimizations of reactants, intermediates, and products were performed within density functional theory using the Perdew–Burke–Ernzerhof (PBE) exchange correlation functional [39] and the projector augmented wave (PAW) method [38, 40]. A planewave energy cutoff of 450 eV was employed. The semi-core 2*p* electrons of magnesium are explicitly included in the valence configuration to improve accuracy [41].

**Fig. 1:**
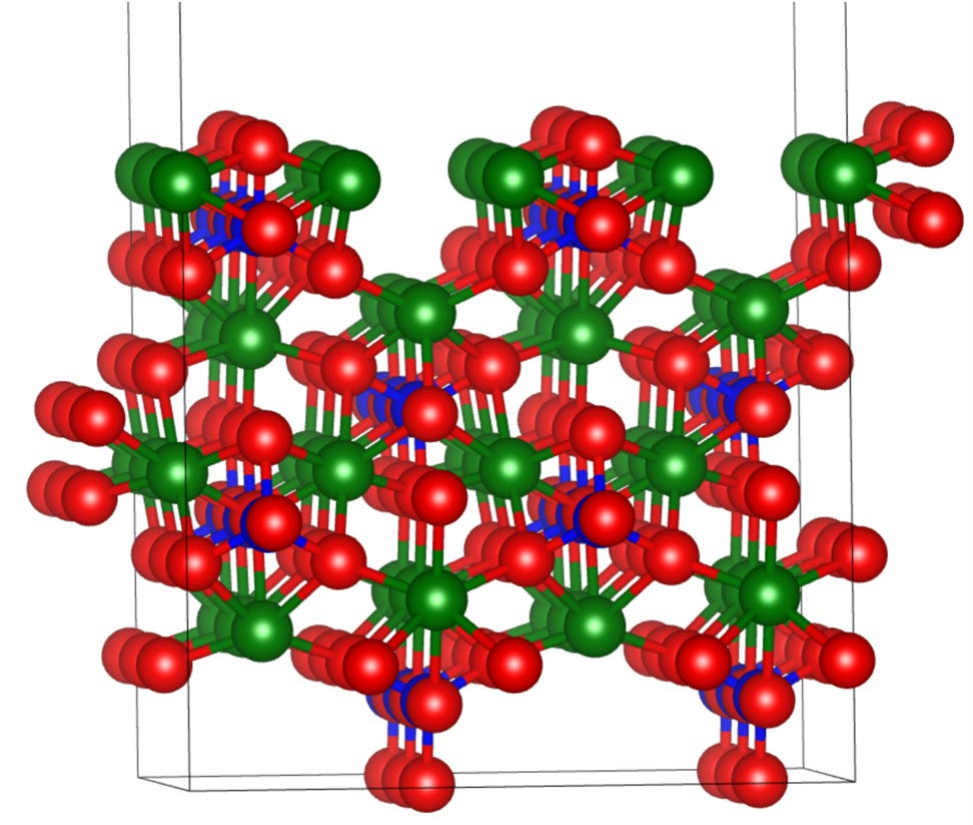
A side-view of the (010) surface of olivine. The red, green, and blue spheres represent oxygen, magnesium, and silicon atoms, respectively. The topmost atoms are surface atoms. The thin lines represent the boundary of the supercell

A (3 × 2) supercell of the forsterite (010) surface, the most stable crystallographic surface of the magnesium-rich olivine endmember was used as the surface model (Fig. 1). The slab preserves the bulk SiO_4_ tetrahedral framework and contains 168 atoms, with a vacuum gap of 15 Å applied perpendicular to the surface to avoid spurious periodic interactions. Structural optimizations employed a force convergence criterion of 0.02 eV Å^−1^; atoms in the bottom slab layer were fixed at bulk positions, while all other atoms were fully relaxed. Surface Brillouin-zone sampling was performed using a 2 × 2 × 1 Monkhorst–Pack k-point mesh [42].

The computational protocol employed in this study successfully reproduces the lattice parameters (within 1% of experimental values) and surface characteristics ((010) being the most stable surface) of olivine reported previously [36, 43]. Moreover, the adsorption behavior of water and various organic molecules [44, 45] on the same olivine surface has been investigated using this protocol, further validating its reliability and applicability. As noted, the present work builds upon and extends the findings of an earlier study, [36] offering a consistent framework for exploring surface interactions relevant to prebiotic chemistry. It should be noted that several types of olivine surfaces have been reported and investigated in the literature [46, 47]. Although it would be possible to explore adsorption and synthesis processes across all such surfaces and using very different methodologies, the present study focuses exclusively on selected surfaces and methods that have been used in previous investigations [36, 43–45, 48, 49].

**Fig. 2:**
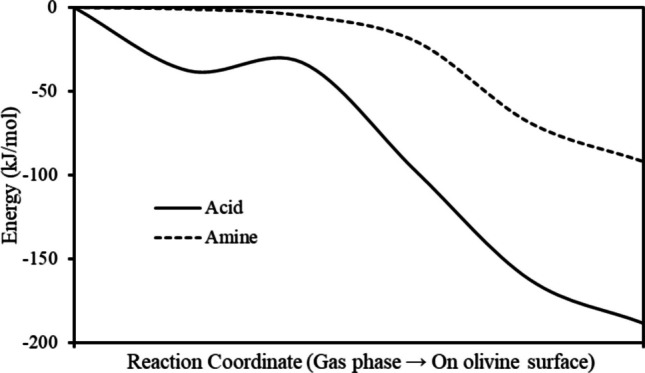
The NEB calculations for formic acid and methyl amine adsorption on the olivine (010) surface

To investigate reaction pathways and energy barriers, I employed NEB method [50]. The minimum energy path (MEP) and associated activation energies were determined using a total of six images, consisting of fully optimized initial and final states connected by four intermediate images. During the NEB optimization, the forces acting perpendicular to the reaction pathway were minimized until they were below 0.05 eV Å^−1^ for all images, ensuring proper convergence of the MEP. For the NEB calculations shown in Fig. 2, the initial states correspond to isolated formic acid or methylamine molecules positioned above the olivine (010) surface at a vertical separation of approximately 5.0 Å from the topmost surface atoms. At this distance, direct chemical interactions are negligible, and the total energy is effectively equivalent to the sum of the clean surface energy and the gas phase molecular energy. This configuration therefore represents a well-defined asymptotic reference state for adsorption. A detailed description of the structural and electronic properties of the modeled olivine surface can be found in prior studies [43, 45].

To assess the thermal stability of the reaction products, ab initio molecular dynamics (AIMD) simulations are performed on the final DFT-optimized structures. These simulations are carried out in the NVT ensemble using the Nosé-Hoover thermostat [51–53]. The time step was set to 0.5 femtoseconds, and the system was equilibrated at 1200 K for a total of 3.5 picoseconds. While the present AIMD simulations are limited to a total trajectory length of 3.5 ps and therefore probe only short time thermal behavior, longer time-scale stability and rare-event processes would require extended simulations or the application of accelerated molecular-dynamics techniques. Such approaches could further clarify long term survivability and temperature dependent behavior of surface bound glycine under more realistic nebular conditions and are a natural direction for future work.

Finally, Bader charge analysis was conducted to evaluate charge redistribution upon adsorption and reaction. The Bader charges are computed using the algorithm developed by Henkelman and co-workers, [54–57] which utilizes the electron density output from VASP to partition charge among atoms.

It is to be noted that while dispersion interactions can be important for weakly bound or physisorbed systems, both formic acid and methylamine bind strongly to the olivine surface through their functional groups, forming short Mg–O and Mg–N bonds characteristic of chemisorption. In such systems, electrostatic and partially covalent interactions dominate, and dispersion contributes only secondarily. Therefore, dispersion correction has not been included in this study.

## Results and discussion

Recent studies have demonstrated that formic acid (HCOOH) and methylamine (CH₃NH₂) exhibit strong adsorption behavior on various mineral surfaces including olivine, magnesite, spinel, and water ice; primarily through their respective functional groups[36]. Building upon these findings, this research focuses on the energetics and kinetics of glycine formation, the simplest amino acid, from precursor organic molecules containing –COOH and –NH₂ functional groups. This transformation is investigated in the context of surface-catalyzed reactions facilitated by olivine, a representative mineral of nebular condensates. As an initial step, I have computed the activation energies associated with the adsorption of formic acid and methylamine individually onto the olivine surface. The reaction coordinate is defined by the NEB minimum energy path connecting the separated molecule-surface configuration to the fully optimized chemisorbed state, with all atomic degrees of freedom relaxed. The results indicate that both molecules adsorb spontaneously, with activation energies effectively calculated as zero relative to their gas phase states, as illustrated in Fig. 2. The shallow local minimum observed along the formic acid adsorption pathway arises from a transient metastable configuration in which the acidic proton of formic acid forms a weak hydrogen bond with a surface oxygen atom, while the carbonyl oxygen simultaneously approaches an undercoordinated surface Mg^2+^ site. This configuration gives rise to a small local minimum along the NEB path (Fig. 2). In contrast, methylamine adsorption proceeds primarily through direct interaction of the nitrogen lone pair with surface Mg2⁺ sites, resulting in a more monotonic energy decrease without an intermediate minimum. The barrierless adsorption process is driven by strong interactions between the functional groups and the mineral surface. Notably, similar behavior has been previously reported for water molecules adsorbing onto the (010) surface of olivine, further supporting the catalytic potential of this mineral in prebiotic chemistry [44]. Because adsorption is barrierless, the initial state does not represent a local minimum but a physically meaningful reference.

The formation of glycine from methylamine and formic acid proceeds via the following reaction:

1 $${\mathrm{CH}}_{3}{\mathrm{NH}}_{2}+\mathrm{H}-\text{COOH }\to {\mathrm{H}}_{2}{\mathrm{NCH}}_{2}\mathrm{COOH}+ {\mathrm{H}}_{2}$$

**Fig. 3:**
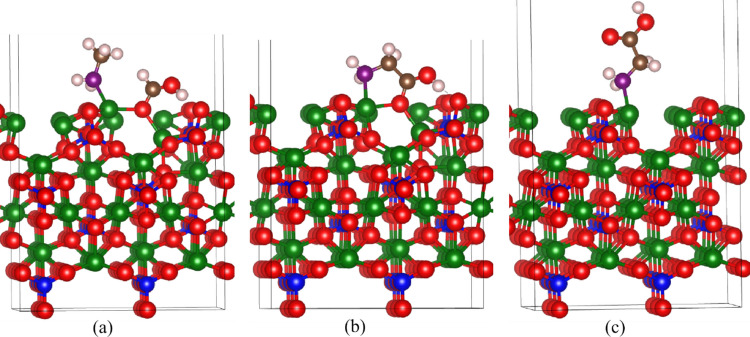
(**a**) Co-adsorption of methyl amine and formic acid, (**b**) glycine formation from (**a**), and (**c**) glycine formation between adsorbed methyl amine and gas phase formic acid. The purple, brown and pinkish-white spheres represent nitrogen, carbon, and hydrogen atoms, respectively. The rest of the presentation is as of Fig. 1

To investigate this transformation, three plausible mechanistic pathways have been explored:

1. Co-adsorption Mechanism (Langmuir–Hinshelwood mechanism): Both formic acid and methylamine are co-adsorbed onto the olivine surface (Fig. 3a), where they subsequently interact to form glycine.
2. Mixed Phase Mechanism (Eley–Rideal mechanism): One reactant (either formic acid or methylamine) is adsorbed on the olivine surface, while the other remains in the gas phase and reacts with the surface-bound species. Both reactants show very similar characteristics, therefore, amine on the surface and formic acid in the gas phase have been presented.
3. Gas Phase Mechanism: Both reactants remain in the gas phase and react without surface mediation.

The final glycine product resulting from the first two surface-mediated mechanisms is illustrated in Figs. 3b and 3c, respectively. It is worth noting that, despite differences in surface materials, Zhao et al. [58] observed adsorption behavior of glycine closely resembling that shown in Fig. 3b, as demonstrated in their combined experimental and computational study.

To quantify the thermodynamics of reaction (1), the reaction energy (ΔE) is calculated using the following expression:

2 $$\Delta \mathrm{E}= {\mathrm{E}}_{\mathrm{surf}-\mathrm{AA}}+{\mathrm{E}}_{\mathrm{H}2}-{\mathrm{E}}_{\mathrm{surf}}-{\mathrm{E}}_{\mathrm{CH}3\mathrm{NH}2 }-{\mathrm{E}}_{\mathrm{HCOOH}}$$

where,

*E*_*surf-AA*_ is the total energy of glycine adsorbed on the olivine surface,

E_*surf*_ is the total energy of the clean olivine surface,

*E*_*H2*_, *E*_*CH3NH2*_ and *E*_*HCOOH*_ are the total energies of hydrogen gas, methylamine, and formic acid, respectively, in the gas phase.

This formulation allows for a plausible comparison of the energetic favorability of glycine formation under different mechanistic conditions, providing insight into the role of mineral surfaces in prebiotic chemistry.

For mechanism (3), the reaction energy is calculated as:

3 $$\Delta \mathrm{E}= {\mathrm{E}}_{\mathrm{AA}}+{\mathrm{E}}_{\mathrm{H}2}-{\mathrm{E}}_{\mathrm{CH}3\mathrm{NH}2 }-{\mathrm{E}}_{\mathrm{HCOOH}}$$

where *E*_*AA*_ is the total energy of glycine in the gas phase.

The calculated reaction energies (ΔE) for glycine formation reveal important insights into the thermodynamic favorability of the proposed mechanisms. For Mechanism (1) where both formic acid and methylamine are co-adsorbed on the olivine surface, the reaction energy is − 283.84 kJ/mol, indicating a highly exothermic and thus energetically favorable process. In contrast, Mechanism (2), in which only one reactant is adsorbed while the other remains in the gas phase, yields a less favorable but still exothermic reaction energy of − 103.24 kJ/mol. These negative values confirm that glycine synthesis is thermodynamically feasible in both surface-mediated scenarios, with Mechanism (1) being significantly more favorable from an energetic standpoint. The enhanced stability of glycine in Mechanism (1) can be attributed to strong interactions between its functional groups and the olivine surface. Specifically, the –COOH and –NH₂ groups of glycine engage in strong ionic interactions with undercoordinated Mg^2^⁺ cations on the olivine surface. The electronegative oxygen and nitrogen atoms in these groups form bonds with the electropositive surface sites, leading to robust adsorption and stabilization of the glycine molecule. In contrast, Mechanism (3), which involves glycine formation entirely in the gas phase is thermodynamically unfavorable, with a calculated reaction energy of + 20.51 kJ/mol. This endothermic nature suggests that, in the absence of a catalytic surface, the formation of glycine from methylamine and formic acid is not spontaneous under standard conditions. It is important to distinguish between the intrinsic chemical transformation energy associated with glycine formation and the overall reaction energies reported for the different mechanisms examined in this study. In principle, the intrinsic energy change associated with the rearrangement of chemical bonds from formic acid and methylamine to glycine and H_2_ is independent of whether the reaction is catalyzed. However, in practice, the total energies calculated here also include adsorption contributions that depend on the binding states of the reactants and products with the olivine surface. For the surface-mediated mechanisms, the reported reaction energies incorporate not only the chemical transformation but also the stabilization arising from adsorption of the reactants and/or the product on the mineral surface. In Mechanism (1), glycine remains strongly adsorbed through interactions involving both its N and O atoms, leading to a large apparent exothermicity. In Mechanism (2), the product is surface bound through fewer functional groups, resulting in a smaller net reaction energy. In contrast, in Mechanism (3), the reaction occurs entirely in the gas phase and does not benefit from surface stabilization, yielding a slightly endothermic reaction energy. Consequently, these reaction energies should not be interpreted as a single intrinsic thermodynamic quantity that can be directly compared across all mechanisms. Rather, they represent the overall energetic balance of each pathway, including adsorption effects. The catalytic role of the olivine surface is therefore more appropriately assessed through the activation barriers, which are referenced to clearly defined initial states specific to each mechanism. The formation of such surface adsorbed amino acids is, in fact, consistent with and supported by previous experimental and observational studies [5–18]. Further, the H_2_ produced in all three mechanisms is in the gas phase.

**Fig. 4:**
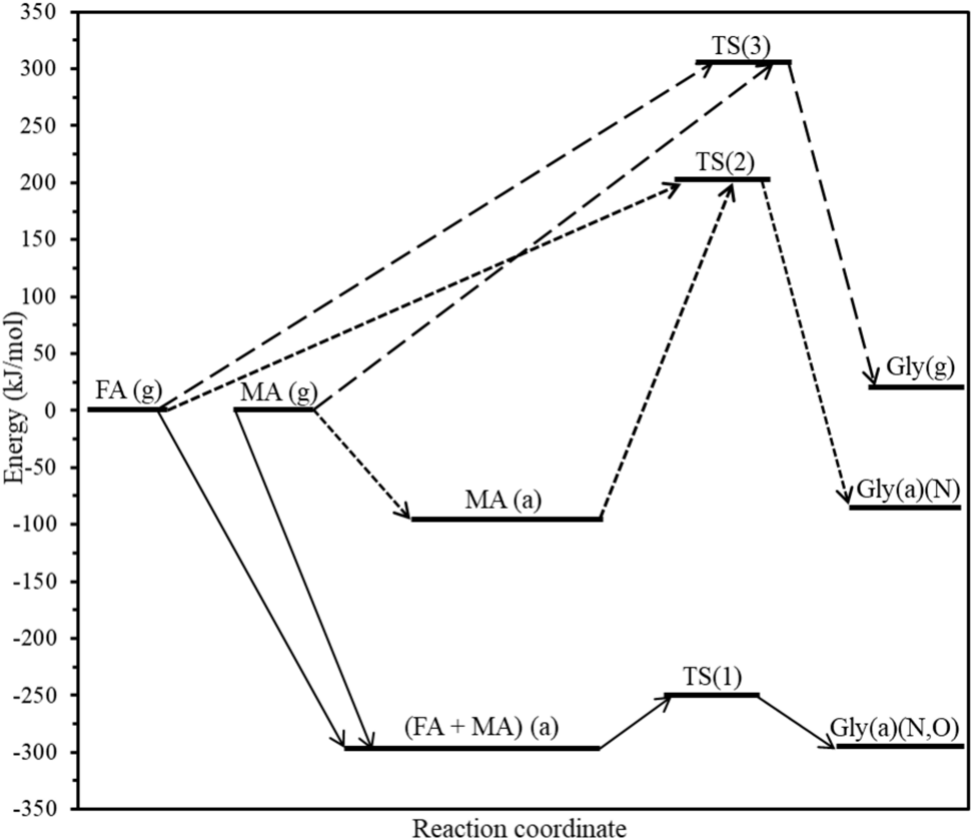
The energy profiles and progress (arrow) for the formation of glycine (Gly) from formic acid (FA) and methyl amine (MA). Mechanism 1: both formic acid and methyl amine are on surface (a) (solid arrow); Mechanism 2: methyl amine on surface (a) and formic acid is gas phase (g) (smaller dashed arrow); Mechanism 3: both methyl amine and formic are in gas phase (g) (longer dashed arrow). TS(1), TS(2) and TS(3) are transition states for mechanisms 1, 2 and 3 respectively. The (a) and (g) after Gly is for glycine on the surface through (N, O) or through (N) bonding and in the gas phase, respectively

Next, I examine the activation barrier energy (Ea) for the formation of glycine. The energy profiles for all three mechanisms are presented in Fig. 4.

Using the Nudged Elastic Band (NEB) method, the activation energy (Eₐ) for glycine formation via Mechanism (1) where a MEP was identified that connects the optimized co-adsorbed reactant configuration to a surface bound glycine product has been calculated at 33.77 kJ/mol. In contrast, Mechanism (2), involving a surface-bound species reacting with a gas phase molecule, exhibits a significantly higher activation barrier of 300.07 kJ/mol. This stark difference highlights the critical role of the olivine surface in modulating the reaction kinetics. The surface not only stabilizes the reactants but also facilitates bond rearrangements by lowering the energy barrier for the transition state. Although Mechanism (3), the gas phase reaction between formic acid and methylamine is thermodynamically unfavorable (ΔE = + 20.51 kJ/mol), its activation energy was also computed for comparison. The result, 310.68 kJ/mol, confirms that this pathway is kinetically hindered and thus highly unlikely under prebiotic conditions. It is to be stressed that the computed NEB pathways should be interpreted as effective surface mediated minimum energy connections between defined initial and final states, rather than as definitive elementary reaction mechanisms.

To the best of my knowledge, no prior studies have investigated the direct synthesis of glycine from formic acid and methylamine. As such, a direct comparison with existing literature is not possible. However, several studies [28–30] have examined glycine formation from other molecular precursors, including both neutral species and free radicals. For instance, Nhlabatsi et al. [29] conducted a computational investigation into glycine formation via a multi-step mechanism. In the initial step, two neutral molecules were involved, and the calculated activation energy ranged from 270 to 300 kJ/mol. In comparison, the activation energy calculated for Mechanism (3) in the present study, also involving two neutral molecules is 311 kJ/mol, which is in close agreement with their findings. This agreement lends confidence to the calculated activation energy for Mechanism (1).

The catalytic effect of the olivine surface can be attributed to strong interactions between the functional groups of the reactants and the surface Mg^2^⁺ sites. Specifically, the nitrogen atom of methylamine and the oxygen atom of formic acid form ionic bonds with undercoordinated magnesium atoms on the surface. These facilitate the distribution of charges and rearrangement bonds along the computed pathway and thereby induce a deficiency in bonding around the carbon atoms, effectively activating them and facilitating the formation of the C–C bond required for glycine synthesis. To be specific, the strong bonds between Mg and N and O, elongates the C-N bond by 0.03 Å in methyl amine C-O in formic acid by 0.2 Å compared to their gas phase. This activation is absent in the gas phase scenario, which explains the significantly higher activation barrier. The extent of carbon activation is further supported by Bader charge analysis: the valence electron count on the carbon atom in formic acid is 1.30 in Mechanism (1), compared to 1.24 in Mechanisms (2) and (3). The higher electron density on carbon in Mechanism (1) is consistent with enhanced surface induced polarization that may contribute to transition state stabilization with the carbon atom of methylamine. A modest increase in electron density on the formic acid carbon in the co-adsorbed mechanism, indicating a higher degree of surface induced polarization relative to the other pathways. While this charge redistribution alone cannot quantitatively explain the differences in activation barriers, it is consistent with enhanced carbon activation arising from strong surface reactant interactions and transition-state stabilization in Mechanism (1). In summary, both thermodynamic and kinetic analysis are consistent with the hypothesis that mineral surfaces such as olivine may facilitate otherwise kinetically inaccessible transformations under prebiotic conditions. The surface not only stabilizes intermediate but also lowers activation barriers, making otherwise improbable reactions feasible under early Earth or nebular conditions.

**Fig. 5:**
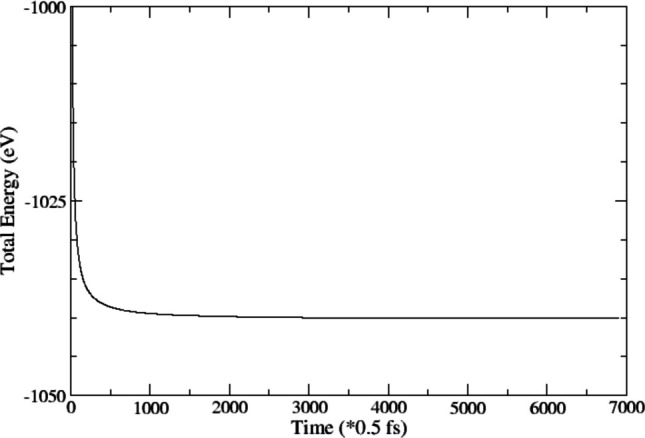
Total energy of the glycine on surface (total energy of the system) over time at 1200 K, which was formed from co-adsorption of formic acid and methyl amine

To assess the thermal stability (short term thermal persistence) of glycine adsorbed on the olivine surface under nebular conditions, ab initio molecular dynamics (AIMD) simulations were performed using the VASP software package. The simulations employed the same supercell configuration used for the optimized glycine product structures. AIMD trajectories were run for 3.5 picoseconds with a 0.5 femtosecond time step, and the system was equilibrated at a high temperature of 1200 K, representative of nebular environments. The primary objective of this study was to determine whether glycine, once formed via Mechanism (1) remains stably adsorbed on the olivine surface at elevated temperatures. Significant desorption or decomposition events would be expected to manifest as abrupt structural changes and corresponding fluctuations in the system’s potential energy. This is crucial for evaluating the plausibility of glycine being synthesized in space and subsequently delivered to the early Earth embedded within an inorganic matrix. As such, gas phase glycine was not considered in these AIMD simulations. The evolution of the system’s total energy over time was monitored to assess the integrity and binding behavior of glycine on the surface. The results, presented in Fig. 5, demonstrate that glycine remains stably adsorbed throughout the simulation duration, indicating that the molecule can withstand high temperature conditions without desorption or decomposition. It is to be noted that these results demonstrate local thermal persistence under high-temperature conditions, but do not address long term stability or rare event processes. Accordingly, conclusions drawn from AIMD are limited to short time behavior, and longer simulations or accelerated dynamics would be required to assess stability on astrophysical timescales.

From Fig. 5, it is clear that glycine molecule does not disintegrate or desorb from the surface at 1200 K temperature. The stability of the glycine molecule on the surface at high temperatures lends the support of experimental and observational studies [5–18] that organic molecules can be intimately associated with inorganic materials.

Finally, it is to be considered that the present results are subject to several limitations, including the use of the PBE functional without explicit dispersion corrections, the absence of hybrid-functional or cluster benchmark calculations, and the idealized representation of the olivine surface. Similarly, the environmental effects such as pressure, wider nebular temperature ranges, explicit gas phase collisions, surface defects, and solvent or ice matrices are not included. Therefore, the results should be interpreted as providing mechanistic insight and qualitative trends rather than definitive kinetic predictions. Investigation of the kinetics including those variables is the motivation for future work.

## Conclusion

It is well established that organic molecules bearing functional groups can strongly adsorb onto meteoritic materials through chemical bonding, particularly with mineral surfaces. In this study, density functional theory (DFT) was employed to explore three plausible mechanisms for the formation of the amino acid glycine, focusing on the catalytic role of the olivine surface. Additionally, the thermal stability of glycine on olivine was investigated using AIMD simulations at 1200 K, a temperature representative of nebular environments.

The calculations suggest that surface mediated pathways can markedly lower apparent activation barriers relative to gas phase reactions, indicating a plausible catalytic role for olivine. Specifically, the reaction is exothermic, and the activation energy for the most favorable mechanism (co-adsorption of reactants on olivine) is only 33.77 kJ/mol, a barrier that is likely surmountable under conditions present in the early solar nebula. In contrast, glycine formation in the gas phase is highly unfavorable, with a positive reaction energy and an activation barrier exceeding 300 kJ/mol, making it unlikely to occur without surface mediation. The present calculations suggest that olivine surfaces may provide an environment in which otherwise highly unfavorable molecular rearrangements leading to amino acid like structures becoming energetically accessible. Comparable activation energies for the synthesis of amino acids from neutral molecular species have been documented in earlier research.

AIMD simulations at 1200 K demonstrate that glycine formed via the co-adsorption pathway remains surface-bound over picosecond timescales without desorption or decomposition. These results indicate short time thermal persistence under high temperature conditions but do not address long term stability or rare event processes relevant to astrophysical timescales.

Recent studies further reinforce the plausibility of multiple prebiotic pathways for glycine formation. For instance, Jarois et al. proposed a novel aza-Cannizzaro mechanism for glycine synthesis from glyoxylic acid in ammonium-rich aqueous environments, highlighting the diversity of chemical routes available under prebiotic conditions [24]. Additionally, Manna and Pal identified methylenimine (CH₂NH) as a key glycine precursor in star forming regions, supporting the idea that amino acid precursors can form and persist in interstellar environments [20].

These findings align with observational evidence from meteorites and cometary samples. Organic compounds, including amino acids, have been identified in carbonaceous chondrites, which are considered among the most pristine meteorites. These include a wide array of compounds such as carboxylic acids, sulfonic and phosphonic acids, amines, amides, alcohols, aldehydes, ketones, and sugars [59]. Notably, the Rosetta mission’s Philae lander detected a diverse suite of organics on Comet 67P/Churyumov-Gerasimenko, and the Stardust mission returned samples from Comet 81P/Wild 2 containing glycine [60]. Similarly, Antarctic micrometeorites have been found to contain significant amounts of organic material, including amino acids [61].

These insights collectively suggest that glycine and other amino acids could have multiple origins, including both surface mediated synthesis on mineral grains and gas phase or aqueous-phase reactions in space. The catalytic role of olivine, as demonstrated in this study, provides a mechanistic framework for how complex organics could emerge and persist in space environments. This contributes to the broader field of astrobiology, emphasizing the importance of mineral-organic interactions in the chemical evolution that may have led to life.

In conclusion, my DFT and AIMD results suggest that olivine surfaces can plausibly catalyze the formation of amino acids like glycine and stabilize them under nebular conditions. This supports the hypothesis that surface catalyzed synthesis on mineral grains could be a viable endogenous source of prebiotic molecules found in meteorites, comets, and other solar system bodies.

## Supplementary Information

Below is the link to the electronic supplementary material.

Supplementary file1 (DOCX 66 KB)

## Data Availability

No datasets were generated or analysed during the current study.
