# Supplementary material for: A quantum-chemical study of synthesis and stability of glycine on olivine surface
Source: J Mol Model. 2026 May 7;32(6):167. doi: 10.1007/s00894-026-06745-0 (PMC13152909; doi:10.1007/s00894-026-06745-0)
Supplement: Supplementary file 1 — Supplementary file1 (DOCX 66 KB) [file 894_2026_6745_MOESM1_ESM.docx]

**A Quantum-Chemical Study of Synthesis and Stability of Glycine on Olivine Surface**

Abu Asaduzzaman

School of Science, Engineering and Technology, Pennsylvania State University Harrisburg, Middletown, PA 17057, USA

e-mail: [aua1309@psu.edu](mailto:aua1309@psu.edu)

<https://orcid.org/0000-0002-6385-3014>

**Supplementary Material**

POSCAR format of all reactants and products

**Methylamine adsorption: Reactant**

Mg O Si H C N

1.00000000000000

12.0060000000000002 0.0000000000000000 0.0000000000000000

0.0000000000000000 14.3070000000000004 0.0000000000000000

0.0000000000000000 0.0000000000000000 29.3124000000000002

Mg O Si H C N

48 96 24 5 1 1

Selective dynamics

Direct

0.0023585044112000 0.9937154180223791 0.3738670205744222 T T T

0.5022690324237340 0.9938655073022284 0.3739324506332364 T T T

0.0023743719555038 0.3272640240316176 0.3738292795431657 T T T

0.5019936723568738 0.3268350042253881 0.3747340427633135 T T T

0.0019534975385922 0.6611195025840059 0.3738030331133750 T T T

0.5019698448333846 0.6613792028036178 0.3740686235318373 T T T

0.0000091113004820 0.1650768410949199 0.1940703572975428 T T T

0.5000178632925101 0.1650738823834588 0.1940816015604594 T T T

-0.0000668306781659 0.4984020776557824 0.1940664955202971 T T T

0.4998979330320931 0.4984302702437368 0.1940469941780260 T T T

0.9999823417383127 0.8316772520034522 0.1940222782653453 T T T

0.4999858530263857 0.8317317894996288 0.1940526622393708 T T T

0.2481981658991194 0.9939276121252894 0.3738528077034153 T T T

0.7482832647221888 0.9938231533159647 0.3738776300650754 T T T

0.2484055098403014 0.3270353888519731 0.3743134045546135 T T T

0.7485356378360353 0.3268596585363657 0.3741101970499965 T T T

0.2479606826685836 0.6614253300437622 0.3739686732508646 T T T

0.7480352873800330 0.6611366323825384 0.3738964467849267 T T T

0.2502033487120295 0.1650781557024796 0.1940217017556233 T T T

0.7501901803322744 0.1650573541179114 0.1940703535058407 T T T

0.2501853147610757 0.4984421664533453 0.1940661957321494 T T T

0.7500856692660360 0.4983953264218143 0.1940857084325284 T T T

0.2502790768149065 0.8317248926797031 0.1940239240069546 T T T

0.7502329447980883 0.8317058698981368 0.1940350635114594 T T T

0.1250000000000000 0.3300334015516881 0.1152112286609110 F F F

0.6250000000000000 0.3300334015516881 0.1152112286609110 F F F

0.1250000000000000 0.6633667348850238 0.1152112286609110 F F F

0.6250000000000000 0.6633667348850238 0.1152112286609110 F F F

0.1250000000000000 0.9967000682183524 0.1152112286609110 F F F

0.6250000000000000 0.9967000682183524 0.1152112286609110 F F F

0.3750000000000000 0.1633667348850238 0.0959918555628363 F F F

0.8750000000000000 0.1633667348850238 0.0959918555628363 F F F

0.3750000000000000 0.4967000682183524 0.0959918555628363 F F F

0.8750000000000000 0.4967000682183524 0.0959918555628363 F F F

0.3750000000000000 0.8300334015516881 0.0959918555628363 F F F

0.8750000000000000 0.8300334015516881 0.0959918555628363 F F F

0.3751780838282409 0.9985189470226168 0.2746722419555587 T T T

0.8752263406849535 0.9984204276250430 0.2746451209729399 T T T

0.3750525807804518 0.3315720913118418 0.2748273038005417 T T T

0.8750066207026480 0.3316560884930597 0.2747380414054924 T T T

0.3751262461619135 0.6648125509244535 0.2748167835996089 T T T

0.8751657678691314 0.6649116375630472 0.2747012400286930 T T T

0.1255109768008902 0.1726376105777362 0.2900796821655224 T T T

0.6253386436278202 0.1726308392244011 0.2901774638662977 T T T

0.1250921177844711 0.5061558399291987 0.2901379435321376 T T T

0.6246800233746764 0.5061433878447341 0.2901420873471118 T T T

0.1252675214295711 0.8391657774935219 0.2899975608101122 T T T

0.6252025943364145 0.8391592968007283 0.2900335018355100 T T T

0.0171000009162086 0.0927333371077097 0.0754446512056361 F F F

0.5171000009162086 0.0927333371077097 0.0754446512056361 F F F

0.0171000009162086 0.4260666704410454 0.0754446512056361 F F F

0.5171000009162086 0.4260666704410454 0.0754446512056361 F F F

0.0171000009162086 0.7594000037743740 0.0754446512056361 F F F

0.5171000009162086 0.7594000037743740 0.0754446512056361 F F F

0.4838732714429482 0.2574531718793436 0.1391759452301500 T T T

0.9838378942136401 0.2574667270842804 0.1391721139550513 T T T

0.4838149815972832 0.5908392783659435 0.1391825307318624 T T T

0.9838187078735440 0.5908058755277686 0.1391938883336232 T T T

0.4839447494542993 0.9241707138549616 0.1392007534929431 T T T

0.9839256595970198 0.9241548430532788 0.1391896905016846 T T T

0.2648860771975038 0.2329306545084309 0.3179328384987433 T T T

0.7646365183183590 0.2326907003450894 0.3180362113900627 T T T

0.2642998110903547 0.5662071183672557 0.3181490970412263 T T T

0.7639870662780786 0.5662507272619112 0.3180189251788004 T T T

0.2645085480448247 0.8996726181665112 0.3177499366392578 T T T

0.7644103072878929 0.8995793161308695 0.3177782551472519 T T T

0.2331669317230930 0.0715134820899312 0.2562487416761646 T T T

0.7331844929557955 0.0714933236652103 0.2563057381951510 T T T

0.2331834460060027 0.4050256398539757 0.2563753153896144 T T T

0.7329988028617443 0.4049896907733274 0.2563597290350682 T T T

0.2331941404839016 0.7381946876655846 0.2562285592582645 T T T

0.7331612008403011 0.7382110268356603 0.2562125450739530 T T T

0.4863521619324967 0.2328332673574144 0.3182687159004026 T T T

0.9861663027665675 0.2326734389425764 0.3179015987994963 T T T

0.4856598969895879 0.5663506592222900 0.3181628304771917 T T T

0.9854707660159434 0.5663657116616435 0.3178654686236769 T T T

0.4861021826385687 0.8996219087025952 0.3178414677520702 T T T

0.9859769664643548 0.8994656232708294 0.3177435692859375 T T T

0.0170706073623715 0.0714917847438643 0.2562314897114107 T T T

0.5170769254835552 0.0715385575243935 0.2562798839647046 T T T

0.0170109341604883 0.4050020026429746 0.2563252112764436 T T T

0.5168483137144489 0.4050131459936962 0.2563444920191995 T T T

0.0170593850365172 0.7382671581066120 0.2561803791759785 T T T

0.5170424549929210 0.7382056744812125 0.2562602656391892 T T T

0.2328999990837914 0.0927333371077097 0.0754446512056361 F F F

0.7328999990837914 0.0927333371077097 0.0754446512056361 F F F

0.2328999990837914 0.4260666704410454 0.0754446512056361 F F F

0.7328999990837914 0.4260666704410454 0.0754446512056361 F F F

0.2328999990837914 0.7594000037743740 0.0754446512056361 F F F

0.7328999990837914 0.7594000037743740 0.0754446512056361 F F F

0.2662939601591021 0.2574882620458117 0.1391529394759831 T T T

0.7662136461482898 0.2574476933888925 0.1391661112133048 T T T

0.2662019495912066 0.5908557374077898 0.1392042904941238 T T T

0.7661761225764557 0.5908080314499420 0.1391963501524464 T T T

0.2663336874741217 0.9241893595190730 0.1391797186701766 T T T

0.7663203865664455 0.9241697666319652 0.1391620956887245 T T T

0.1250000000000000 0.2554999946180203 0.0502498420463695 F F F

0.6250000000000000 0.2554999946180203 0.0502498420463695 F F F

0.1250000000000000 0.5888333279513489 0.0502498420463695 F F F

0.6250000000000000 0.5888333279513489 0.0502498420463695 F F F

0.1250000000000000 0.9221666612846846 0.0502498420463695 F F F

0.6250000000000000 0.9221666612846846 0.0502498420463695 F F F

0.1251341396124767 0.0719621955952562 0.1788942013757922 T T T

0.6251464337741861 0.0719145212113914 0.1789336906570868 T T T

0.1250975210605265 0.4051952693202476 0.1789919176351762 T T T

0.6250047512445464 0.4051715244989451 0.1789973776272000 T T T

0.1251567123934055 0.7385568489858644 0.1788449530406452 T T T

0.6250407038700920 0.7385858764495048 0.1788801974511171 T T T

0.3750447353756881 0.4209716360149858 0.1650343322933294 T T T

0.8749922691238715 0.4209617946989349 0.1650552071088843 T T T

0.3751067300358467 0.7543067341800563 0.1650176789894002 T T T

0.8750508039974304 0.7542245114136039 0.1650385842606325 T T T

0.3751321709043959 0.0876137939515910 0.1651459556662191 T T T

0.8751107361952973 0.0875861664699610 0.1651451279156201 T T T

0.3749342463669184 0.2406999471041580 0.3940495096641619 T T T

0.8757391686022944 0.2411782942597429 0.3938846404742243 T T T

0.3748736227015028 0.5754439079934824 0.3940853254676266 T T T

0.8750567979610451 0.5749327967088805 0.3938447269555936 T T T

0.3751787102091899 0.9077461219979420 0.3937554870663028 T T T

0.8753128753120467 0.9076068832306677 0.3937297233909828 T T T

0.3752588605361432 0.0730918165707689 0.3453761904687350 T T T

0.8754223296436004 0.0731233569463221 0.3454344833363344 T T T

0.3753696051786877 0.4068026795023851 0.3460976589886752 T T T

0.8750333101765855 0.4066167734172799 0.3454622882425576 T T T

0.3750743404363771 0.7400810067199679 0.3452454370555664 T T T

0.8749890071770809 0.7399926645042609 0.3451830895522456 T T T

0.3750869739352388 0.2576476815325943 0.2154862802108713 T T T

0.8750570487548446 0.2576021889160671 0.2154639474163477 T T T

0.3750443742555895 0.5909571305623749 0.2154832604576101 T T T

0.8750098203995775 0.5908640458524362 0.2154720061143363 T T T

0.3751337077054813 0.9242430676334512 0.2154690981233401 T T T

0.8750824655112790 0.9241938834784175 0.2154493252250238 T T T

0.1251634865608743 0.9088842158172821 0.2289056760828660 T T T

0.6251295218211643 0.9088975996796901 0.2289383115766910 T T T

0.1251762789261780 0.2423036773539594 0.2290500495808035 T T T

0.6250555802383967 0.2422680755524531 0.2291027378376209 T T T

0.1250947661347915 0.5756084471003189 0.2289439726375663 T T T

0.6249749160687810 0.5755993832309245 0.2289519609851409 T T T

0.1250000000000000 0.0936333333333366 0.0000000000000000 F F F

0.6250000000000000 0.0936333333333366 0.0000000000000000 F F F

0.1250000000000000 0.4269666666666652 0.0000000000000000 F F F

0.6250000000000000 0.4269666666666652 0.0000000000000000 F F F

0.1250000000000000 0.7603000000000009 0.0000000000000000 F F F

0.6250000000000000 0.7603000000000009 0.0000000000000000 F F F

0.1250000000000000 0.1423333253652075 0.0512282806593802 F F F

0.6250000000000000 0.1423333253652075 0.0512282806593802 F F F

0.1250000000000000 0.4756666586985361 0.0512282806593802 F F F

0.6250000000000000 0.4756666586985361 0.0512282806593802 F F F

0.1250000000000000 0.8089999920318718 0.0512282806593802 F F F

0.6250000000000000 0.8089999920318718 0.0512282806593802 F F F

0.3750845041867122 0.3074346706017603 0.1630632895849413 T T T

0.8750127271644773 0.3074263784522970 0.1630500493183294 T T T

0.3750350614355797 0.6407807670531435 0.1630667239697283 T T T

0.8749947789610250 0.6407091306236459 0.1630675508177938 T T T

0.3751348130809386 0.9740926583584023 0.1630781572208642 T T T

0.8750993975695710 0.9740698991881359 0.1630692882592136 T T T

0.3754002793538047 0.1873896910869331 0.3441702160128515 T T T

0.8755214709511506 0.1873786164329324 0.3441088295010454 T T T

0.3750125702932616 0.5211046335191475 0.3444843328381253 T T T

0.8748450944645829 0.5209692428617821 0.3441240386959112 T T T

0.3752311112010714 0.8543333501461918 0.3439025207278812 T T T

0.8751864400792019 0.8542273792123659 0.3438698134075062 T T T

0.1251079608259040 0.0229042911445280 0.2300859007126567 T T T

0.6251402841516563 0.0229182397490849 0.2301333404866372 T T T

0.1251215375699479 0.3563532742455025 0.2302234703141721 T T T

0.6249530387994349 0.3563218193865640 0.2302294227539081 T T T

0.1251381788569929 0.6896319546499444 0.2300599320588100 T T T

0.6250615987600753 0.6896207588049027 0.2300864974239228 T T T

0.3482765555107376 0.3347163583634967 0.5963330253866517 T T T

0.4093690935089807 0.4164570494329679 0.6347204478047894 T T T

0.3530926601523305 0.4548466770309347 0.5816786315291460 T T T

0.5536021862696202 0.4413660864884593 0.5743163979610624 T T T

0.5497645646636063 0.3297233889906130 0.5884091694081922 T T T

0.3996459634432657 0.3981591927276369 0.5983018029363226 T T T

0.5046196159155493 0.3826411545220423 0.5737132344321706 T T T

**Methylamine adsorption: Product**

Mg O Si H C N

1.00000000000000

12.0060000000000002 0.0000000000000000 0.0000000000000000

0.0000000000000000 14.3070000000000004 0.0000000000000000

0.0000000000000000 0.0000000000000000 29.3124000000000002

Mg O Si H C N

48 96 24 5 1 1

Selective dynamics

Direct

0.0030340919170126 0.9932839673166930 0.3737022419023365 T T T

0.5026642137936295 0.9938089086157812 0.3735325332307219 T T T

0.0034147862447122 0.3277598343536647 0.3732394365345641 T T T

0.4912608097680920 0.3279944074908657 0.3828625394140728 T T T

0.0023200606568633 0.6613243228549266 0.3739161465528747 T T T

0.5027694183278095 0.6619733011294099 0.3739341156028189 T T T

0.0000775743573552 0.1649660310607095 0.1940192850224659 T T T

0.5001761880290878 0.1651692354320980 0.1941018327941243 T T T

0.0002073471633111 0.4984044642887921 0.1940577265790752 T T T

0.5000443106611558 0.4983926940112760 0.1939658415307116 T T T

0.9999195888577667 0.8315734708852299 0.1940334685720043 T T T

0.4999380562997653 0.8319255298109669 0.1940751636706516 T T T

0.2480560219106043 0.9939439346178457 0.3736036168985492 T T T

0.7482030512236346 0.9940493167457315 0.3740015050143636 T T T

0.2440325008760234 0.3259502675321481 0.3719057669426464 T T T

0.7519236958872628 0.3260028916265687 0.3717671779379009 T T T

0.2474673011097715 0.6619963205819320 0.3741408255872209 T T T

0.7476785083442667 0.6604596514819392 0.3742368502415057 T T T

0.2503987006652001 0.1650224226741106 0.1940191574301591 T T T

0.7501243266531493 0.1650151628964618 0.1942739378697777 T T T

0.2504033828210456 0.4985421239398610 0.1940716097246164 T T T

0.7501722307788351 0.4983432271220956 0.1940545729761166 T T T

0.2502389599550694 0.8316917619447731 0.1940552957503694 T T T

0.7500984101475714 0.8318349481177021 0.1940356784806895 T T T

0.1250000000000000 0.3300334015516881 0.1152112286609110 F F F

0.6250000000000000 0.3300334015516881 0.1152112286609110 F F F

0.1250000000000000 0.6633667348850238 0.1152112286609110 F F F

0.6250000000000000 0.6633667348850238 0.1152112286609110 F F F

0.1250000000000000 0.9967000682183524 0.1152112286609110 F F F

0.6250000000000000 0.9967000682183524 0.1152112286609110 F F F

0.3750000000000000 0.1633667348850238 0.0959918555628363 F F F

0.8750000000000000 0.1633667348850238 0.0959918555628363 F F F

0.3750000000000000 0.4967000682183524 0.0959918555628363 F F F

0.8750000000000000 0.4967000682183524 0.0959918555628363 F F F

0.3750000000000000 0.8300334015516881 0.0959918555628363 F F F

0.8750000000000000 0.8300334015516881 0.0959918555628363 F F F

0.3748878336346610 -0.0014386284195352 0.2747604706895506 T T T

0.8753994004517119 -0.0018844122283316 0.2747107919272575 T T T

0.3753987149911678 0.3317090431687595 0.2750855672848723 T T T

0.8761054515102134 0.3317476846685393 0.2742463365517774 T T T

0.3749078048987368 0.6647656457550543 0.2749097001741508 T T T

0.8752414335586580 0.6650923052800111 0.2746787842563683 T T T

0.1271339620345905 0.1722770086673454 0.2897971483660323 T T T

0.6251063151066309 0.1725392554318774 0.2908880708603705 T T T

0.1252317335396469 0.5060121454796260 0.2903508530319377 T T T

0.6243997828878363 0.5068070164082503 0.2898644964703438 T T T

0.1250601578424027 0.8389413159592815 0.2900846300815625 T T T

0.6252784614953326 0.8390427384475543 0.2900401412174730 T T T

0.0171000009162086 0.0927333371077097 0.0754446512056361 F F F

0.5171000009162086 0.0927333371077097 0.0754446512056361 F F F

0.0171000009162086 0.4260666704410454 0.0754446512056361 F F F

0.5171000009162086 0.4260666704410454 0.0754446512056361 F F F

0.0171000009162086 0.7594000037743740 0.0754446512056361 F F F

0.5171000009162086 0.7594000037743740 0.0754446512056361 F F F

0.4840989797545175 0.2575256499762804 0.1392498552200050 T T T

0.9837789918343630 0.2574808334791425 0.1391327313702822 T T T

0.4840136917915479 0.5908709830881033 0.1393008089080454 T T T

0.9838276986774356 0.5907706544543846 0.1392293252828506 T T T

0.4839250187446009 0.9241945221729616 0.1392139737615579 T T T

0.9837581225298107 0.9241494619806023 0.1392317459008164 T T T

0.2697339693097472 0.2336032392373881 0.3158694593684049 T T T

0.7659716164530334 0.2325655090330666 0.3176809134813268 T T T

0.2644781409443806 0.5658061884970483 0.3187398465870315 T T T

0.7638494038364649 0.5660591279552207 0.3179700563023171 T T T

0.2647595446692111 0.8993622321345637 0.3175199784363003 T T T

0.7638319568217258 0.8992827595228259 0.3180595917659756 T T T

0.2330732000251465 0.0713554831667970 0.2560995876640192 T T T

0.7332106251182003 0.0714312162583082 0.2565835295886617 T T T

0.2334360113483977 0.4051182822984794 0.2565247587595493 T T T

0.7329578617843142 0.4048318844581366 0.2567597868140499 T T T

0.2330367605196893 0.7379233165944248 0.2563578829180322 T T T

0.7331808651442467 0.7382262246241188 0.2561652589139138 T T T

0.4913996943523048 0.2338031205443495 0.3225036683198467 T T T

0.9872431211350907 0.2321927859389354 0.3174081833410505 T T T

0.4856929171344675 0.5655462026033966 0.3186868727778721 T T T

0.9854587478221155 0.5668513249759741 0.3176195386594995 T T T

0.4863831457883989 0.8991291621398003 0.3178303721578299 T T T

0.9853641722583317 0.8989692985423613 0.3176428846596136 T T T

0.0170544577404766 0.0713455795403374 0.2560830524245175 T T T

0.5170307499890053 0.0721220922584468 0.2567280053004355 T T T

0.0173636521144526 0.4050744087822183 0.2561898158449414 T T T

0.5168297961512197 0.4049932691481453 0.2567154508406352 T T T

0.0169628750373406 0.7381694595529100 0.2561128108101767 T T T

0.5171938310591534 0.7378902254915480 0.2564308546948863 T T T

0.2328999990837914 0.0927333371077097 0.0754446512056361 F F F

0.7328999990837914 0.0927333371077097 0.0754446512056361 F F F

0.2328999990837914 0.4260666704410454 0.0754446512056361 F F F

0.7328999990837914 0.4260666704410454 0.0754446512056361 F F F

0.2328999990837914 0.7594000037743740 0.0754446512056361 F F F

0.7328999990837914 0.7594000037743740 0.0754446512056361 F F F

0.2665334603199349 0.2574838458133536 0.1392768620978926 T T T

0.7659707050292671 0.2574512663732036 0.1392849878794577 T T T

0.2664056305168043 0.5908976807706796 0.1392641877257371 T T T

0.7661092216147974 0.5907812174931211 0.1392719791406312 T T T

0.2663280843617847 0.9242070146047083 0.1392404011337830 T T T

0.7660995702557865 0.9242067657821187 0.1392296112206179 T T T

0.1250000000000000 0.2554999946180203 0.0502498420463695 F F F

0.6250000000000000 0.2554999946180203 0.0502498420463695 F F F

0.1250000000000000 0.5888333279513489 0.0502498420463695 F F F

0.6250000000000000 0.5888333279513489 0.0502498420463695 F F F

0.1250000000000000 0.9221666612846846 0.0502498420463695 F F F

0.6250000000000000 0.9221666612846846 0.0502498420463695 F F F

0.1251827495255802 0.0718865832458729 0.1788018083235195 T T T

0.6249711007391233 0.0719948070098013 0.1792152261825099 T T T

0.1255593429043922 0.4052283297984635 0.1789765582586905 T T T

0.6250539359594081 0.4049532150651517 0.1793203450641992 T T T

0.1252077336417845 0.7384093839779271 0.1788838182127586 T T T

0.6248815309869500 0.7387507569669967 0.1789032153942485 T T T

0.3752345229811123 0.4210065841296107 0.1651014909700312 T T T

0.8750566299297403 0.4209033397950754 0.1651450430789718 T T T

0.3751637373325196 0.7542918870764359 0.1651467336683095 T T T

0.8749579994265472 0.7541840704946258 0.1650749624146117 T T T

0.3753160870226180 0.0876294778551792 0.1651950420834361 T T T

0.8749320130142345 0.0876038061334504 0.1652150731014539 T T T

0.3657898408391019 0.2368128733791187 0.3949552596478872 T T T

0.8767288638713681 0.2409934404170783 0.3933704100005685 T T T

0.3755161267250472 0.5756818250918102 0.3948295289182013 T T T

0.8754171242823936 0.5740428417284237 0.3938181476909978 T T T

0.3752419759388848 0.9077888143631041 0.3936158467745153 T T T

0.8753849763566599 0.9076936145025803 0.3938205705023280 T T T

0.3754864305161493 0.0726095411070067 0.3446192783003415 T T T

0.8759604069296135 0.0725876918828747 0.3451473084571547 T T T

0.3752882304365980 0.4066667628089846 0.3477065605860216 T T T

0.8757896459062617 0.4067367245754747 0.3447374262040661 T T T

0.3751831027389029 0.7398023551384364 0.3452099327241114 T T T

0.8744862341888904 0.7396863588881737 0.3454200954472776 T T T

0.3755722751973908 0.2577800917708968 0.2156167954373693 T T T

0.8751411582870950 0.2574906925651427 0.2154122845877330 T T T

0.3752287827582310 0.5908856596893352 0.2155744215285861 T T T

0.8750687638513233 0.5907457514418488 0.2154933077662716 T T T

0.3752028831095626 0.9243366526290833 0.2155064072386946 T T T

0.8749304350539188 0.9241402193558441 0.2154891570561884 T T T

0.1251359097037303 0.9086873330495812 0.2289049065277075 T T T

0.6247876799951838 0.9091261277308641 0.2291087642638164 T T T

0.1254620616321313 0.2423499010658871 0.2289289702193217 T T T

0.6247212080458756 0.2421884207072137 0.2296225878223717 T T T

0.1253696418467780 0.5754460356433256 0.2290500475887613 T T T

0.6249640333487078 0.5756752451130848 0.2288530642956378 T T T

0.1250000000000000 0.0936333333333366 0.0000000000000000 F F F

0.6250000000000000 0.0936333333333366 0.0000000000000000 F F F

0.1250000000000000 0.4269666666666652 0.0000000000000000 F F F

0.6250000000000000 0.4269666666666652 0.0000000000000000 F F F

0.1250000000000000 0.7603000000000009 0.0000000000000000 F F F

0.6250000000000000 0.7603000000000009 0.0000000000000000 F F F

0.1250000000000000 0.1423333253652075 0.0512282806593802 F F F

0.6250000000000000 0.1423333253652075 0.0512282806593802 F F F

0.1250000000000000 0.4756666586985361 0.0512282806593802 F F F

0.6250000000000000 0.4756666586985361 0.0512282806593802 F F F

0.1250000000000000 0.8089999920318718 0.0512282806593802 F F F

0.6250000000000000 0.8089999920318718 0.0512282806593802 F F F

0.3753444072318237 0.3074584219099052 0.1631569438082674 T T T

0.8749479553963264 0.3073557673980591 0.1630186079558777 T T T

0.3752010055924473 0.6407983131790165 0.1631508497719978 T T T

0.8749890531446414 0.6406714730256514 0.1630971604982153 T T T

0.3751556542750716 0.9741237490430302 0.1631008738765379 T T T

0.8749412640476316 0.9740867340894446 0.1631147220651430 T T T

0.3764825861573011 0.1869575667021140 0.3444726985717300 T T T

0.8771554146155519 0.1866200713965307 0.3436551867478319 T T T

0.3749688865111617 0.5209547967722487 0.3451425090490143 T T T

0.8751744267885974 0.5212190033062121 0.3438306517302860 T T T

0.3752849870229307 0.8541327718806332 0.3438166312769755 T T T

0.8747745810033034 0.8539530377646163 0.3440433545235139 T T T

0.1250052913197427 0.0227024030760856 0.2299618343295459 T T T

0.6249450258622672 0.0231801831006892 0.2304687800176624 T T T

0.1256082301407726 0.3563889878714975 0.2301968197442297 T T T

0.6248974623156681 0.3562228722782037 0.2305745873656186 T T T

0.1251052096863497 0.6894729745273381 0.2300949331543055 T T T

0.6250696271184268 0.6897119601445864 0.2300530207231116 T T T

0.3465675409998281 0.3344953698805997 0.4764609095307671 T T T

0.4127711842794101 0.4148136877927192 0.5144542883892314 T T T

0.3546838641218479 0.4561564887603795 0.4620794624426443 T T T

0.5515647852833158 0.4408266753731376 0.4531520871024937 T T T

0.5513365291349218 0.3321210678888616 0.4705685320354810 T T T

0.3982435515759593 0.3976177920413057 0.4783913611326235 T T T

0.5043128053912210 0.3806737503484514 0.4531086615103451 T T T

**Formic acid adsorption: Reactant**

Mg O Si H C

1.00000000000000

12.0060000000000002 0.0000000000000000 0.0000000000000000

0.0000000000000000 14.3070000000000004 0.0000000000000000

0.0000000000000000 0.0000000000000000 29.3124000000000002

Mg O Si H C

48 98 24 2 1

Selective dynamics

Direct

0.0020337185605196 0.9944021293649146 0.3738972804837047 T T T

0.5019530191149432 0.9944684226747625 0.3739054888235468 T T T

0.0020727862280458 0.3289718749494993 0.3737949123267567 T T T

0.5016771705833165 0.3286078794588946 0.3740344984222426 T T T

0.0022486985528889 0.6622120075408430 0.3734085989254569 T T T

0.5019605761461360 0.6619766612495026 0.3737530528360032 T T T

0.9998597701307931 0.1657045535960871 0.1940131304725364 T T T

0.4998972400806232 0.1657315002381546 0.1940428279753647 T T T

0.9998273646298016 0.4989951657668288 0.1939780434704418 T T T

0.4998771823367663 0.4990015166710590 0.1939881644396023 T T T

0.9998539095011980 0.8323439096882655 0.1939916580128897 T T T

0.4998744648034276 0.8323502354953782 0.1939922795939510 T T T

0.2479336258824808 0.9943680657295376 0.3738895348889528 T T T

0.7480385695578697 0.9944839789222891 0.3738979945788738 T T T

0.2479902069884441 0.3289688365534071 0.3737012712205808 T T T

0.7484926129548032 0.3286620517847191 0.3743793263401690 T T T

0.2479413600187406 0.6621347482041997 0.3734615451485377 T T T

0.7482156877414083 0.6619796197660812 0.3738585189003287 T T T

0.2501084830001158 0.1657036981121599 0.1940129430309465 T T T

0.7500902205364801 0.1657316445946957 0.1940363544494345 T T T

0.2501121644585471 0.4989974637853103 0.1939645778919686 T T T

0.7501003359829682 0.4990037678525714 0.1939715051716992 T T T

0.2501213577119391 0.8323417081651575 0.1939877600870632 T T T

0.7501149870557255 0.8323569489008251 0.1939911890406976 T T T

0.1250000000000000 0.3300334015516881 0.1152112286609110 F F F

0.6250000000000000 0.3300334015516881 0.1152112286609110 F F F

0.1250000000000000 0.6633667348850238 0.1152112286609110 F F F

0.6250000000000000 0.6633667348850238 0.1152112286609110 F F F

0.1250000000000000 0.9967000682183524 0.1152112286609110 F F F

0.6250000000000000 0.9967000682183524 0.1152112286609110 F F F

0.3750000000000000 0.1633667348850238 0.0959918555628363 F F F

0.8750000000000000 0.1633667348850238 0.0959918555628363 F F F

0.3750000000000000 0.4967000682183524 0.0959918555628363 F F F

0.8750000000000000 0.4967000682183524 0.0959918555628363 F F F

0.3750000000000000 0.8300334015516881 0.0959918555628363 F F F

0.8750000000000000 0.8300334015516881 0.0959918555628363 F F F

0.3749659253869594 -0.0006386416482450 0.2745354811602693 T T T

0.8749976259829035 -0.0006218171110845 0.2745368711507251 T T T

0.3750332273290390 0.3323971769800105 0.2746455901127919 T T T

0.8749477428698319 0.3323658718956985 0.2746985846834299 T T T

0.3750113922697287 0.6660284331671483 0.2745111061254703 T T T

0.8749711928247830 0.6660025027861083 0.2745394147470777 T T T

0.1249107554373132 0.1736195830945170 0.2900402209794216 T T T

0.6250372854258278 0.1736510669642213 0.2901561460984656 T T T

0.1250082768257891 0.5068614052393805 0.2898899833920768 T T T

0.6251010370819106 0.5069174108996535 0.2899586647748090 T T T

0.1249955121505597 0.8400718099611674 0.2899219207835427 T T T

0.6250124722702731 0.8401319526529726 0.2899128351068176 T T T

0.0171000009162086 0.0927333371077097 0.0754446512056361 F F F

0.5171000009162086 0.0927333371077097 0.0754446512056361 F F F

0.0171000009162086 0.4260666704410454 0.0754446512056361 F F F

0.5171000009162086 0.4260666704410454 0.0754446512056361 F F F

0.0171000009162086 0.7594000037743740 0.0754446512056361 F F F

0.5171000009162086 0.7594000037743740 0.0754446512056361 F F F

0.4837995949806033 0.2578607001454662 0.1391280169556983 T T T

0.9837482207259027 0.2578593906128849 0.1391228898574183 T T T

0.4837995630269262 0.5912499443877751 0.1390909207800045 T T T

0.9837379907945171 0.5912444736661409 0.1390890345940636 T T T

0.4837788656774534 0.9245732576629192 0.1391522584876512 T T T

0.9837560517061119 0.9245757654131367 0.1391503279826177 T T T

0.2642410581205373 0.2336514265045463 0.3179094036099477 T T T

0.7641245795227998 0.2336549498213649 0.3182203677478042 T T T

0.2644181284365711 0.5672954180106088 0.3175409029622127 T T T

0.7640612429687339 0.5672926782708737 0.3178561391997610 T T T

0.2643458137839143 0.9005838269081137 0.3175692433460572 T T T

0.7642273163713195 0.9007126415619805 0.3175982259706213 T T T

0.2330238743380724 0.0725143104002352 0.2561534669090191 T T T

0.7330277533331583 0.0725810722714534 0.2562107449465777 T T T

0.2330469262756697 0.4057127290609290 0.2561518695028774 T T T

0.7330862156822319 0.4057477196310276 0.2562038098425313 T T T

0.2330295744471712 0.7391591281261859 0.2560116347050114 T T T

0.7330362464315130 0.7391767383815936 0.2560417924930813 T T T

0.4857487874357012 0.2335909608939553 0.3180836030055313 T T T

0.9856117261299601 0.2337308908766403 0.3179106980612871 T T T

0.4860013165845112 0.5673217892407488 0.3177608646559634 T T T

0.9856176558515801 0.5672553353434345 0.3175352481254615 T T T

0.4857818499928290 0.9006629479187059 0.3176112843812502 T T T

0.9856616006488637 0.9006302150409562 0.3175629055651780 T T T

0.0168969614310368 0.0725143699527819 0.2561395269585786 T T T

0.5169245692619480 0.0725475035032247 0.2562031179604541 T T T

0.0169052267780205 0.4056913072929443 0.2561701948932321 T T T

0.5169519332161007 0.4057404704182987 0.2562069632538152 T T T

0.0169527013547363 0.7391511519584565 0.2560258595112722 T T T

0.5169578305729678 0.7391824249073832 0.2560340299075449 T T T

0.2328999990837914 0.0927333371077097 0.0754446512056361 F F F

0.7328999990837914 0.0927333371077097 0.0754446512056361 F F F

0.2328999990837914 0.4260666704410454 0.0754446512056361 F F F

0.7328999990837914 0.4260666704410454 0.0754446512056361 F F F

0.2328999990837914 0.7594000037743740 0.0754446512056361 F F F

0.7328999990837914 0.7594000037743740 0.0754446512056361 F F F

0.2662325548117734 0.2578623158917228 0.1391205411760104 T T T

0.7661912664579483 0.2578574177854396 0.1391296875211187 T T T

0.2662492438084810 0.5912491714860162 0.1390827950250113 T T T

0.7661938975496658 0.5912565745525786 0.1390902500665210 T T T

0.2662363953004028 0.9245763899827100 0.1391508668974015 T T T

0.7662157201253038 0.9245731527080453 0.1391506848290570 T T T

0.1250000000000000 0.2554999946180203 0.0502498420463695 F F F

0.6250000000000000 0.2554999946180203 0.0502498420463695 F F F

0.1250000000000000 0.5888333279513489 0.0502498420463695 F F F

0.6250000000000000 0.5888333279513489 0.0502498420463695 F F F

0.1250000000000000 0.9221666612846846 0.0502498420463695 F F F

0.6250000000000000 0.9221666612846846 0.0502498420463695 F F F

0.1249884650346327 0.0726145855024507 0.1787985071502680 T T T

0.6249971876332863 0.0726434216647162 0.1788387443396488 T T T

0.1249604883626060 0.4057548206579141 0.1788159671106303 T T T

0.6249832290918317 0.4057447000969451 0.1788523516525123 T T T

0.1249837070473968 0.7392481847609208 0.1786902852626579 T T T

0.6250011373804123 0.7392449117161400 0.1787110915810561 T T T

0.3750087157810156 0.4214823983280999 0.1649089260075529 T T T

0.8749560705319491 0.4214881629426197 0.1649019179177401 T T T

0.3750040691875702 0.7547722333199082 0.1649387323429569 T T T

0.8749846486297445 0.7547754605267830 0.1649411482410760 T T T

0.3750248338495571 0.0881023231149309 0.1650753397399164 T T T

0.8749623835670781 0.0881030526172959 0.1650728437082474 T T T

0.3746154007926598 0.2427170156450515 0.3938977829311146 T T T

0.8755544377419736 0.2426430965213626 0.3939912587627205 T T T

0.3747182447844838 0.5760163324590125 0.3935383160980326 T T T

0.8756154056876820 0.5762138165647920 0.3935725728906126 T T T

0.3749944557577537 0.9081113860287149 0.3935800907790405 T T T

0.8749890388745919 0.9081041379794900 0.3935755515321390 T T T

0.3748930529092622 0.0742023054719386 0.3457322067225920 T T T

0.8750993260194982 0.0742345043197423 0.3457483429245554 T T T

0.3751909102407757 0.4077941956637081 0.3451990200775106 T T T

0.8748553946523839 0.4078191560988382 0.3453868260682268 T T T

0.3751411929971600 0.7408419876729891 0.3448307166183354 T T T

0.8749000822732588 0.7408711530006854 0.3448007543848101 T T T

0.3750111805729980 0.2583373288522536 0.2154010116471227 T T T

0.8749672068750822 0.2583446768582714 0.2154106944925476 T T T

0.3750172635644186 0.5915229504942778 0.2153561818596132 T T T

0.8749499007699619 0.5915261550718167 0.2153637882609531 T T T

0.3750044861036654 0.9248865420295982 0.2154264211445281 T T T

0.8749799346272046 0.9248953644687490 0.2154261499575366 T T T

0.1249876620125879 0.9097632471526895 0.2288570019156247 T T T

0.6250021714494486 0.9098102380431033 0.2288671407374267 T T T

0.1249698100845661 0.2429812297608813 0.2289501392064695 T T T

0.6250172663553951 0.2430184842680940 0.2290322005371226 T T T

0.1249898655393312 0.5763697240841905 0.2287547574815723 T T T

0.6250139368916794 0.5763868621058440 0.2288133640636938 T T T

0.1250000000000000 0.0936333333333366 0.0000000000000000 F F F

0.6250000000000000 0.0936333333333366 0.0000000000000000 F F F

0.1250000000000000 0.4269666666666652 0.0000000000000000 F F F

0.6250000000000000 0.4269666666666652 0.0000000000000000 F F F

0.1250000000000000 0.7603000000000009 0.0000000000000000 F F F

0.6250000000000000 0.7603000000000009 0.0000000000000000 F F F

0.6782762193339870 0.4439910985087049 0.5440633378523302 T T T

0.7234221199311260 0.4773202467933057 0.6179508410727970 T T T

0.1250000000000000 0.1423333253652075 0.0512282806593802 F F F

0.6250000000000000 0.1423333253652075 0.0512282806593802 F F F

0.1250000000000000 0.4756666586985361 0.0512282806593802 F F F

0.6250000000000000 0.4756666586985361 0.0512282806593802 F F F

0.1250000000000000 0.8089999920318718 0.0512282806593802 F F F

0.6250000000000000 0.8089999920318718 0.0512282806593802 F F F

0.3750159280387013 0.3079360955381980 0.1629555185003600 T T T

0.8749684767469232 0.3079370550131660 0.1629628861633066 T T T

0.3750187300690480 0.6412575129456880 0.1629395811629938 T T T

0.8749695854346989 0.6412618667873387 0.1629461726841876 T T T

0.3750124672014969 0.9745829981798798 0.1630139248828609 T T T

0.8749795209292559 0.9745847684237613 0.1630110377165952 T T T

0.3748613001645603 0.1884997695773391 0.3442507258195189 T T T

0.8750827372991243 0.1885494285339599 0.3443104857441078 T T T

0.3750777694280699 0.5221184015695000 0.3438251431452092 T T T

0.8750528061431683 0.5221471644679206 0.3439019963741525 T T T

0.3750611460743573 0.8550909568457032 0.3436454900867403 T T T

0.8749463736864469 0.8551144029310087 0.3436291828378579 T T T

0.1249791110690308 0.0237742471222602 0.2300315366496619 T T T

0.6249853902497847 0.0238288134249033 0.2300808032452786 T T T

0.1249635490995776 0.3569963220105407 0.2300587257638322 T T T

0.6250163781956352 0.3570284310168458 0.2301077742814115 T T T

0.1249835557852980 0.6903979941234107 0.2299126868679046 T T T

0.6249975021448353 0.6904125704806168 0.2299378637679731 T T T

0.7591160019493537 0.5317498018842456 0.6036943477042125 T T T

0.6347267338290140 0.3643258132555458 0.5999518727412860 T T T

0.6760504430999127 0.4258320280172017 0.5846268177752217 T T T

**Formic acid adsorption: Product**

Mg O Si H C

1.00000000000000

12.0060000000000002 0.0000000000000000 0.0000000000000000

0.0000000000000000 14.3070000000000004 0.0000000000000000

0.0000000000000000 0.0000000000000000 29.3124000000000002

Mg O Si H C

48 98 24 2 1

Selective dynamics

Direct

0.0005400377518222 0.9945813266614079 0.3745591105568422 T T T

0.5025767616705612 0.9942657205901629 0.3756689053856413 T T T

0.0103994627894641 0.3256341162211564 0.3718020482660250 T T T

0.4917955188398702 0.3349664657442132 0.3669511975330871 T T T

0.0063683165001259 0.6652646371657480 0.3736729974922107 T T T

0.4999654281059623 0.6513751816572909 0.3746875746828864 T T T

0.9997700441993698 0.1648083279398782 0.1941446730397433 T T T

0.5003803952278510 0.1651222064597754 0.1941919487482233 T T T

0.0003830438392812 0.4989923461618668 0.1934452952512170 T T T

0.5008992333028358 0.4989249871141620 0.1948216611562310 T T T

0.0000476684042884 0.8321502321474664 0.1940676841582700 T T T

0.5006371118817576 0.8315911727680602 0.1940239674810863 T T T

0.2466628807005923 0.9938706593411591 0.3743528638443802 T T T

0.7457953206723344 0.9940917761218571 0.3757251796383184 T T T

0.2445658346758973 0.3278548370207157 0.3727430817618553 T T T

0.7646225194929083 0.3417673157664524 0.3766953351534587 T T T

0.2468286677455611 0.6627905570967004 0.3743979741507269 T T T

0.7490470769520864 0.6471059378207750 0.3736941442432077 T T T

0.2503338324042825 0.1646343347663873 0.1941807185317626 T T T

0.7501321882156256 0.1653960943789551 0.1940270435207703 T T T

0.2505650981213725 0.4987677394253351 0.1932598503473732 T T T

0.7501501657949918 0.4988025146366750 0.1948606411045768 T T T

0.2505590567325807 0.8319782013476834 0.1942521608458851 T T T

0.7498722706200160 0.8315924693045890 0.1941022600335942 T T T

0.1250000000000000 0.3300334015516881 0.1152112286609110 F F F

0.6250000000000000 0.3300334015516881 0.1152112286609110 F F F

0.1250000000000000 0.6633667348850238 0.1152112286609110 F F F

0.6250000000000000 0.6633667348850238 0.1152112286609110 F F F

0.1250000000000000 0.9967000682183524 0.1152112286609110 F F F

0.6250000000000000 0.9967000682183524 0.1152112286609110 F F F

0.3750000000000000 0.1633667348850238 0.0959918555628363 F F F

0.8750000000000000 0.1633667348850238 0.0959918555628363 F F F

0.3750000000000000 0.4967000682183524 0.0959918555628363 F F F

0.8750000000000000 0.4967000682183524 0.0959918555628363 F F F

0.3750000000000000 0.8300334015516881 0.0959918555628363 F F F

0.8750000000000000 0.8300334015516881 0.0959918555628363 F F F

0.3751656075487959 0.9972248240088505 0.2752046735283588 T T T

0.8750701135775106 0.9973608765605705 0.2753382733186888 T T T

0.3717317516108667 0.3318332547814479 0.2733570209490515 T T T

0.8789756221949612 0.3323475213026636 0.2743834055374936 T T T

0.3749716684675130 0.6663082316298832 0.2741096706314712 T T T

0.8753036207648417 0.6668901197235509 0.2737385205737507 T T T

0.1227918187734413 0.1719235532455504 0.2906791029897609 T T T

0.6259711352731372 0.1715025921981040 0.2909077435550613 T T T

0.1279806641259272 0.5066956403782842 0.2885542783450216 T T T

0.6268865272660563 0.5010152621766916 0.2933204639754685 T T T

0.1242977992882765 0.8388205638817174 0.2908975814866900 T T T

0.6242033139403470 0.8413221828887335 0.2899036679180237 T T T

0.0171000009162086 0.0927333371077097 0.0754446512056361 F F F

0.5171000009162086 0.0927333371077097 0.0754446512056361 F F F

0.0171000009162086 0.4260666704410454 0.0754446512056361 F F F

0.5171000009162086 0.4260666704410454 0.0754446512056361 F F F

0.0171000009162086 0.7594000037743740 0.0754446512056361 F F F

0.5171000009162086 0.7594000037743740 0.0754446512056361 F F F

0.4845086919872259 0.2574560298771967 0.1393351442401991 T T T

0.9837579613755525 0.2575542848727881 0.1394507771981829 T T T

0.4851534916792941 0.5907310606735527 0.1396014336368845 T T T

0.9831730006826193 0.5911509260767212 0.1388762009066056 T T T

0.4844016984263284 0.9242207298880390 0.1394783481826405 T T T

0.9835716100257644 0.9244917256500499 0.1394946744410286 T T T

0.2665710047726024 0.2315365255098993 0.3170901990396756 T T T

0.7534894606566592 0.2360138170968363 0.3248189493010410 T T T

0.2632993108363359 0.5711968082059863 0.3159747859438871 T T T

0.7728749327080473 0.5662680185168261 0.3159698338168783 T T T

0.2657535633768460 0.8973493428765499 0.3186595626029330 T T T

0.7612022368236238 0.8985608077981772 0.3196486159707410 T T T

0.2333112602790472 0.0709047499525271 0.2567395507265612 T T T

0.7333693531621673 0.0718382424759811 0.2572878745148430 T T T

0.2333575814804387 0.4060655830736965 0.2549929264521544 T T T

0.7340682794240699 0.4033292109024439 0.2574405695167123 T T T

0.2334230543939351 0.7390690113852023 0.2558473295123417 T T T

0.7328514880144844 0.7389168709983451 0.2572288193224341 T T T

0.4888141117093634 0.2333549934286942 0.3188370116162493 T T T

0.9770718281529629 0.2317769439304029 0.3162064901014957 T T T

0.4839963901588868 0.5675276570682971 0.3172996215350702 T T T

0.9930802539958061 0.5715482154889736 0.3162111235816920 T T T

0.4866605484861743 0.8993486394458262 0.3192175712479683 T T T

0.9818663054162559 0.8974444139044366 0.3185985816487193 T T T

0.0169112318913291 0.0707477042198922 0.2568945580080626 T T T

0.5171510938070973 0.0712979721857939 0.2570329187565479 T T T

0.0179765791926170 0.4064641222616565 0.2553249000280555 T T T

0.5179123349662960 0.4029762925839205 0.2579693628258298 T T T

0.0172587591320879 0.7389457489464328 0.2559241294480802 T T T

0.5173646680109568 0.7388647402596136 0.2571539307308744 T T T

0.2328999990837914 0.0927333371077097 0.0754446512056361 F F F

0.7328999990837914 0.0927333371077097 0.0754446512056361 F F F

0.2328999990837914 0.4260666704410454 0.0754446512056361 F F F

0.7328999990837914 0.4260666704410454 0.0754446512056361 F F F

0.2328999990837914 0.7594000037743740 0.0754446512056361 F F F

0.7328999990837914 0.7594000037743740 0.0754446512056361 F F F

0.2665784782236401 0.2574821760555882 0.1392629481358324 T T T

0.7660326538200987 0.2577471293968138 0.1393843179791105 T T T

0.2674968479027281 0.5911112089119822 0.1388758097418841 T T T

0.7653983885677096 0.5907484700551092 0.1395567763907431 T T T

0.2665659070696567 0.9244020082237677 0.1395366431363214 T T T

0.7658023210442890 0.9241981717932721 0.1394781096451627 T T T

0.1250000000000000 0.2554999946180203 0.0502498420463695 F F F

0.6250000000000000 0.2554999946180203 0.0502498420463695 F F F

0.1250000000000000 0.5888333279513489 0.0502498420463695 F F F

0.6250000000000000 0.5888333279513489 0.0502498420463695 F F F

0.1250000000000000 0.9221666612846846 0.0502498420463695 F F F

0.6250000000000000 0.9221666612846846 0.0502498420463695 F F F

0.1249081670747432 0.0717546852855330 0.1794069917107178 T T T

0.6254683104799500 0.0721327278077598 0.1794850445216980 T T T

0.1252405417329659 0.4052322560347579 0.1781049933698139 T T T

0.6251800473995757 0.4051866703129803 0.1800405229716925 T T T

0.1251687730765771 0.7393421950648129 0.1785695184183912 T T T

0.6252260076965140 0.7374961974660865 0.1798342967743262 T T T

0.3760975219310800 0.4208949151656493 0.1653943739716061 T T T

0.8747804779485031 0.4211283880637180 0.1654228784531134 T T T

0.3756976586205971 0.7543819913073607 0.1652021150769050 T T T

0.8747676100342702 0.7544683991011910 0.1651801028646937 T T T

0.3755573609370806 0.0875534450428789 0.1654238031395031 T T T

0.8746816109875876 0.0876534762683443 0.1654026295824681 T T T

0.3776321017291476 0.2469573681133931 0.3930403965044184 T T T

0.8803123470058239 0.2468277075610588 0.3952161810020848 T T T

0.3713081342878928 0.5693894627213217 0.3929546245981995 T T T

0.8841770672594378 0.5684383640274333 0.3925344374387623 T T T

0.3746355876211016 0.9086667347680077 0.3950360349287595 T T T

0.8737338607827094 0.9082467746952866 0.3951634548971403 T T T

0.3761708046979469 0.0741249631607749 0.3468873383263138 T T T

0.8710606839570143 0.0754262107487269 0.3478028967810530 T T T

0.3672382597936386 0.4080249028131001 0.3407847411142794 T T T

0.8861307751570537 0.4074189267939605 0.3411462438150762 T T T

0.3801639011107164 0.7394017131878741 0.3482229787874586 T T T

0.8695180717729775 0.7385070851544333 0.3483329689841983 T T T

0.3752921103984893 0.2571874313448155 0.2153564643463706 T T T

0.8749804613775298 0.2575537007576719 0.2156503803536335 T T T

0.3754591277693151 0.5910992391054526 0.2154426213347261 T T T

0.8754568275694719 0.5912998677781045 0.2153682425677550 T T T

0.3757979395777445 0.9240747178786095 0.2157533185363254 T T T

0.8744732672534329 0.9242918734564688 0.2157702806105959 T T T

0.1249479950052969 0.9086345637652498 0.2295563480387168 T T T

0.6254090407572215 0.9094208140207767 0.2291313507135267 T T T

0.1249151248147887 0.2425475957928949 0.2293625420651608 T T T

0.6258625613434121 0.2415275692242367 0.2296846165215537 T T T

0.1258188870020523 0.5763621932669932 0.2278336207318617 T T T

0.6255541085238417 0.5748371495576337 0.2310268446759993 T T T

0.1250000000000000 0.0936333333333366 0.0000000000000000 F F F

0.6250000000000000 0.0936333333333366 0.0000000000000000 F F F

0.1250000000000000 0.4269666666666652 0.0000000000000000 F F F

0.6250000000000000 0.4269666666666652 0.0000000000000000 F F F

0.1250000000000000 0.7603000000000009 0.0000000000000000 F F F

0.6250000000000000 0.7603000000000009 0.0000000000000000 F F F

0.6209522133509850 0.4195743356462086 0.3677180237537130 T T T

0.7403784037903061 0.4522413381964019 0.4258333444140736 T T T

0.1250000000000000 0.1423333253652075 0.0512282806593802 F F F

0.6250000000000000 0.1423333253652075 0.0512282806593802 F F F

0.1250000000000000 0.4756666586985361 0.0512282806593802 F F F

0.6250000000000000 0.4756666586985361 0.0512282806593802 F F F

0.1250000000000000 0.8089999920318718 0.0512282806593802 F F F

0.6250000000000000 0.8089999920318718 0.0512282806593802 F F F

0.3755352351127766 0.3073914660183550 0.1629684348308529 T T T

0.8749087900656886 0.3076211789031429 0.1632028141682959 T T T

0.3760784514520705 0.6409056130953797 0.1630026222366983 T T T

0.8745300091219806 0.6409538646566008 0.1629271605876536 T T T

0.3755669626692993 0.9740557003061905 0.1633478703549862 T T T

0.8745950518990129 0.9741420072288339 0.1633313764037613 T T T

0.3762568074147826 0.1878960752350996 0.3443861576324079 T T T

0.8699149534205993 0.1897550651930834 0.3463204135071263 T T T

0.3707077147350671 0.5219827125144764 0.3415732689173385 T T T

0.8856907835102297 0.5210303661084934 0.3403524790483558 T T T

0.3764498724305025 0.8538583821789626 0.3456404009678887 T T T

0.8717301830170889 0.8532196249811025 0.3458380441707805 T T T

0.1251603934831931 0.0225962252322140 0.2306037619217801 T T T

0.6253634298144189 0.0234262459742990 0.2306875183142099 T T T

0.1251934465213897 0.3566097424362993 0.2292803912034692 T T T

0.6257697520953581 0.3556057168271240 0.2310776156647908 T T T

0.1254248797223495 0.6903342339113703 0.2297018537086203 T T T

0.6251772125806632 0.6889772821786624 0.2312272153472457 T T T

0.8039812891236348 0.4983425128146050 0.4213963942470853 T T T

0.5736584771982631 0.4876647669929337 0.4280666970219292 T T T

0.6425518828961336 0.4869914155392189 0.4031699925430872 T T T

**Glycine formation, Mechanism 1: Reactant**

Mg O Si H C N

1.00000000000000

12.0060000000000002 0.0000000000000000 0.0000000000000000

0.0000000000000000 14.3070000000000004 0.0000000000000000

0.0000000000000000 0.0000000000000000 29.3124000000000002

Mg O Si H C N

48 98 24 7 2 1

Selective dynamics

Direct

0.0004664226973255 0.9879121161873057 0.3740894945524916 T T T

0.4993373216268373 0.9933450833384563 0.3750396870234184 T T T

0.0001960722595010 0.3296245029017018 0.3738634485270903 T T T

0.4539308322997260 0.3483421327750685 0.4006608153902665 T T T

0.9933467968888244 0.6625032976518793 0.3745602990274363 T T T

0.4931129102803506 0.6679741140080239 0.3756118424013368 T T T

0.9984673169291483 0.1647271786912747 0.1941526276915280 T T T

0.4985408091379187 0.1637287169042314 0.1950154057206526 T T T

0.0011144862587190 0.4988012053460230 0.1936676405715378 T T T

0.5002105633783506 0.4960345436434379 0.1950205044280206 T T T

0.0014993119644217 0.8328609876856433 0.1935017124012901 T T T

0.5009146368582138 0.8321678436320628 0.1937095201539199 T T T

0.2433516054953710 0.9986411098729201 0.3738142377948475 T T T

0.7411315998960543 0.9882476777186296 0.3747382867722964 T T T

0.2307234408573808 0.3252315133307666 0.3682148376017341 T T T

0.7158322116213502 0.3538285290222813 0.3415366557800131 T T T

0.2328800755448986 0.6688473683909343 0.3746568912629186 T T T

0.7355083328715393 0.6596719118402895 0.3771790654422323 T T T

0.2491984252772476 0.1644848711600314 0.1945265907063501 T T T

0.7473258655041144 0.1644136317466085 0.1950444495748665 T T T

0.2516175283853881 0.4987464982042646 0.1952888152913851 T T T

0.7482366178193504 0.5032754408600664 0.1907580998430743 T T T

0.2512469197349301 0.8316757500302246 0.1942480271918975 T T T

0.7507974579161977 0.8322191570849412 0.1943837144289141 T T T

0.1250000000000000 0.3300334015516881 0.1152112286609110 F F F

0.6250000000000000 0.3300334015516881 0.1152112286609110 F F F

0.1250000000000000 0.6633667348850238 0.1152112286609110 F F F

0.6250000000000000 0.6633667348850238 0.1152112286609110 F F F

0.1250000000000000 0.9967000682183524 0.1152112286609110 F F F

0.6250000000000000 0.9967000682183524 0.1152112286609110 F F F

0.3750000000000000 0.1633667348850238 0.0959918555628363 F F F

0.8750000000000000 0.1633667348850238 0.0959918555628363 F F F

0.3750000000000000 0.4967000682183524 0.0959918555628363 F F F

0.8750000000000000 0.4967000682183524 0.0959918555628363 F F F

0.3750000000000000 0.8300334015516881 0.0959918555628363 F F F

0.8750000000000000 0.8300334015516881 0.0959918555628363 F F F

0.3721679319120092 0.9974947210135656 0.2752442755141510 T T T

0.8733611743534104 0.9955716047999815 0.2754836055413579 T T T

0.3734745723041383 0.3280987619937583 0.2761407137181924 T T T

0.8879967405477130 0.3293454846913675 0.2736074005914761 T T T

0.3736864203797139 0.6682935428212468 0.2754427752000900 T T T

0.8804499150598698 0.6692757224916621 0.2737354355626850 T T T

0.1227445397038139 0.1695267107826033 0.2910311288162067 T T T

0.6159048539089348 0.1630251123122600 0.2937396713670978 T T T

0.1131653334411222 0.5073655927842722 0.2914070750757521 T T T

0.6020227396088947 0.5161727356306328 0.2905435315937062 T T T

0.1221673819791459 0.8391860964970994 0.2905858306007220 T T T

0.6204374009451752 0.8381379973240948 0.2912972048412132 T T T

0.0171000009162086 0.0927333371077097 0.0754446512056361 F F F

0.5171000009162086 0.0927333371077097 0.0754446512056361 F F F

0.0171000009162086 0.4260666704410454 0.0754446512056361 F F F

0.5171000009162086 0.4260666704410454 0.0754446512056361 F F F

0.0171000009162086 0.7594000037743740 0.0754446512056361 F F F

0.5171000009162086 0.7594000037743740 0.0754446512056361 F F F

0.4860594098577846 0.2586186689613517 0.1409056081389038 T T T

0.9819338577344708 0.2585826859609275 0.1400723759822652 T T T

0.4869373762356426 0.5896310421104618 0.1409388228301684 T T T

0.9849089438431589 0.5908482006203253 0.1396558674989793 T T T

0.4836021158576911 0.9243381623636315 0.1393801744963299 T T T

0.9830644006762156 0.9251758964779062 0.1393157151902787 T T T

0.2667659543253469 0.2332450364280216 0.3161180306911630 T T T

0.7473192390394225 0.2217102432580609 0.3293510972077866 T T T

0.2461873703048850 0.5660277370133932 0.3219263098469184 T T T

0.7337782726176511 0.5428745341240576 0.3295177299047777 T T T

0.2600520288999842 0.9027555485277713 0.3170691643129844 T T T

0.7592178708025861 0.8976028335099103 0.3176442548388554 T T T

0.2317962649627414 0.0718779028245175 0.2560321242010909 T T T

0.7315239654886968 0.0704440076549982 0.2573077434938771 T T T

0.2343779452457981 0.4051564453282671 0.2572864432616173 T T T

0.7222557922389790 0.3851327952779086 0.2726654116802164 T T T

0.2340108047503258 0.7388741715569808 0.2557853622309029 T T T

0.7347921792512647 0.7356772898681307 0.2569291881732058 T T T

0.4868098771614569 0.2362736296460777 0.3212104207132219 T T T

0.9696579842363587 0.2319663843436399 0.3166051710840352 T T T

0.4666084646187381 0.5700486410220480 0.3187911712177498 T T T

0.9615666119415329 0.5650565817957066 0.3151071307293632 T T T

0.4803035919966008 0.8988145931913927 0.3177819693086154 T T T

0.9794177969169970 0.8954110706430961 0.3170523767516282 T T T

0.0156258656875347 0.0708235774803480 0.2567044465393916 T T T

0.5150862571766848 0.0686863607383860 0.2572090737006474 T T T

0.0185959724887295 0.4061945819870438 0.2563112875013957 T T T

0.5124426745770033 0.4063950194324486 0.2592347995456145 T T T

0.0187140959181786 0.7389877120098279 0.2556900192157042 T T T

0.5185912497680848 0.7364379705230772 0.2571759992542862 T T T

0.2328999990837914 0.0927333371077097 0.0754446512056361 F F F

0.7328999990837914 0.0927333371077097 0.0754446512056361 F F F

0.2328999990837914 0.4260666704410454 0.0754446512056361 F F F

0.7328999990837914 0.4260666704410454 0.0754446512056361 F F F

0.2328999990837914 0.7594000037743740 0.0754446512056361 F F F

0.7328999990837914 0.7594000037743740 0.0754446512056361 F F F

0.2669505976997487 0.2576691951782970 0.1398395305779377 T T T

0.7634526609576265 0.2580606718245797 0.1402451568306201 T T T

0.2685042184777759 0.5902848570748422 0.1400464764782279 T T T

0.7679097211591758 0.5926586676782427 0.1382647916071779 T T T

0.2658504373300468 0.9240406357619058 0.1396435497886509 T T T

0.7652623701654043 0.9243834041250445 0.1398024473570896 T T T

0.1250000000000000 0.2554999946180203 0.0502498420463695 F F F

0.6250000000000000 0.2554999946180203 0.0502498420463695 F F F

0.1250000000000000 0.5888333279513489 0.0502498420463695 F F F

0.6250000000000000 0.5888333279513489 0.0502498420463695 F F F

0.1250000000000000 0.9221666612846846 0.0502498420463695 F F F

0.6250000000000000 0.9221666612846846 0.0502498420463695 F F F

0.1233509118362517 0.0723541261848492 0.1791221238418431 T T T

0.6235729620343773 0.0710408457659861 0.1798844554310541 T T T

0.1266529805954124 0.4053586722101554 0.1795198197330531 T T T

0.6330720107570336 0.4035871253410881 0.1863121528795917 T T T

0.1261183920495885 0.7383371773205109 0.1785708823834629 T T T

0.6264129269926397 0.7380513586850654 0.1794058869113239 T T T

0.3766213148891058 0.4205008629769493 0.1663494514878572 T T T

0.8733970419836995 0.4212874486515900 0.1668766818349819 T T T

0.3767934070259618 0.7534978394347456 0.1658530451285002 T T T

0.8761072619681990 0.7550161759169061 0.1654552740026870 T T T

0.3743374071616302 0.0876033686277572 0.1654929440137309 T T T

0.8731931841622335 0.0881619158026086 0.1657457737494872 T T T

0.3575730935967291 0.2396840668259158 0.3959743366742856 T T T

0.8988742599128083 0.2221642700915231 0.3980585197291311 T T T

0.3639564690855459 0.5808506471336347 0.3966136847747087 T T T

0.8700245051958838 0.5747574639418217 0.3955712570873198 T T T

0.3686870119390229 0.9089554304877321 0.3937775512125952 T T T

0.8707571156496524 0.9020293873266272 0.3935898161668465 T T T

0.3746400310615148 0.0758307547384171 0.3464360821044371 T T T

0.8721033768889646 0.0671909222073195 0.3451667752979246 T T T

0.3651400777116689 0.4114448504338442 0.3515416524527255 T T T

0.8713982832754663 0.4024601197491407 0.3476655166740156 T T T

0.3653480456268111 0.7416465843934225 0.3448252581753387 T T T

0.8648328663045158 0.7356557578677267 0.3436009352604508 T T T

0.3741709678452779 0.2569120187146640 0.2163492818592933 T T T

0.8717886564172350 0.2579657996790046 0.2161589574833447 T T T

0.3764558639303925 0.5906009596443365 0.2167485590983334 T T T

0.8734935290902049 0.5910535551721261 0.2152837893456420 T T T

0.3752827126918622 0.9240271508284877 0.2156580347649655 T T T

0.8749940219081737 0.9241647473607433 0.2156581669616189 T T T

0.1244959047544897 0.9087641357156754 0.2289424458920238 T T T

0.6246586865553392 0.9072853211436764 0.2295431013828471 T T T

0.1225578767403206 0.2426620069518040 0.2302637736801075 T T T

0.6197367940176883 0.2385192436058867 0.2326028592033973 T T T

0.1239911242976316 0.5758014121701128 0.2296944703041864 T T T

0.6265736768940531 0.5747756643550560 0.2296687693235138 T T T

0.1250000000000000 0.0936333333333366 0.0000000000000000 F F F

0.6250000000000000 0.0936333333333366 0.0000000000000000 F F F

0.1250000000000000 0.4269666666666652 0.0000000000000000 F F F

0.6250000000000000 0.4269666666666652 0.0000000000000000 F F F

0.1250000000000000 0.7603000000000009 0.0000000000000000 F F F

0.6250000000000000 0.7603000000000009 0.0000000000000000 F F F

0.6206419034224190 0.3663021773528030 0.3992395561983907 T T T

0.7611926363570363 0.3248243377019854 0.4471339061432671 T T T

0.1250000000000000 0.1423333253652075 0.0512282806593802 F F F

0.6250000000000000 0.1423333253652075 0.0512282806593802 F F F

0.1250000000000000 0.4756666586985361 0.0512282806593802 F F F

0.6250000000000000 0.4756666586985361 0.0512282806593802 F F F

0.1250000000000000 0.8089999920318718 0.0512282806593802 F F F

0.6250000000000000 0.8089999920318718 0.0512282806593802 F F F

0.3755293929768261 0.3070144108546997 0.1640208706406359 T T T

0.8724789642663042 0.3076777350181027 0.1635912506335595 T T T

0.3771908256533545 0.6400236581675778 0.1640637009239932 T T T

0.8764877473097692 0.6419114267855145 0.1629243226353253 T T T

0.3747786281199904 0.9740655468450474 0.1633046999006331 T T T

0.8741942850216806 0.9745594370330898 0.1633516269570510 T T T

0.3758239901523893 0.1903028065986486 0.3455502527182046 T T T

0.8681693456583803 0.1802706484091972 0.3454516257989201 T T T

0.3587276229912501 0.5252996747743389 0.3470185245634106 T T T

0.8619802561866959 0.5177222100971712 0.3471141318382623 T T T

0.3685187456212489 0.8560640772671180 0.3439674085253632 T T T

0.8687864905620432 0.8503012611941086 0.3434405168982159 T T T

0.1237268027791854 0.0227007573342883 0.2301929493911061 T T T

0.6240477948393605 0.0213486724718834 0.2308932531221710 T T T

0.1277913657869808 0.3566509680905082 0.2306506604948005 T T T

0.6219329284194750 0.3518764549128249 0.2365169145317893 T T T

0.1265141507590934 0.6899241840953676 0.2297500523662341 T T T

0.6266168247851626 0.6896907940541694 0.2304801428698787 T T T

0.3032660816649907 0.3941603418184789 0.4589096088503503 T T T

0.3755463818710886 0.4883873772105794 0.4471871794746500 T T T

0.4362042606674496 0.3467344740136791 0.5186572446923124 T T T

0.3622696204437775 0.4519366537641743 0.5305392290082618 T T T

0.8389065578906292 0.2471994778948108 0.4181474108079626 T T T

0.5012869127641100 0.4574781235202046 0.5103155362681262 T T T

0.6248669904610180 0.3961963629438884 0.4678028592659196 T T T

0.4225881335424844 0.4189892154010520 0.5073889956493800 T T T

0.6712468906343837 0.3623166012475837 0.4396678271351530 T T T

0.3836989315772918 0.4200117422373342 0.4591104423840028 T T T

**Glycine formation, Mechanism 1: Product**

Mg O Si H C N

1.00000000000000

12.0060000000000002 0.0000000000000000 0.0000000000000000

0.0000000000000000 14.3070000000000004 0.0000000000000000

0.0000000000000000 0.0000000000000000 29.3124000000000002

Mg O Si H C N

48 98 24 7 2 1

Selective dynamics

Direct

0.9981696927402568 0.9881052937853715 0.3749257834649025 T T T

0.4978749307582881 0.9924410932323835 0.3750006808892019 T T T

0.0031388734076854 0.3296202030718983 0.3745206065380671 T T T

0.4681851131186622 0.3299930329651871 0.3867944103416486 T T T

0.9954628633868069 0.6635755318249605 0.3745748551414383 T T T

0.4945009385410349 0.6636701661865982 0.3754399126381773 T T T

0.9986922839280475 0.1642882808121663 0.1936750854097836 T T T

0.4986217688059723 0.1639196058146770 0.1953514192124440 T T T

0.0007796098675108 0.4988663721251335 0.1935813086056282 T T T

0.5004431730804707 0.4964691941324301 0.1946783791306917 T T T

0.0011370370415645 0.8327761446949086 0.1934659159158411 T T T

0.5005537978939617 0.8322408514531375 0.1938334208418378 T T T

0.2406740163141020 0.9942385786811843 0.3742827317198628 T T T

0.7396760572271475 0.9887366639000503 0.3749462224963503 T T T

0.2319591099116213 0.3251608497238609 0.3703505274500900 T T T

0.7250303573061432 0.3586767496442883 0.3388515971899317 T T T

0.2360241884247774 0.6641235172540362 0.3754531240046168 T T T

0.7372439036093242 0.6571398354157331 0.3777117391406658 T T T

0.2494757859606479 0.1642888118447744 0.1948979800337449 T T T

0.7475044911291014 0.1639825187691937 0.1954492107367262 T T T

0.2513374907953548 0.4989047913677506 0.1948951680011277 T T T

0.7484894969448934 0.5023724474114997 0.1911641115231346 T T T

0.2510437088608158 0.8317466436509761 0.1942956980493036 T T T

0.7501899350195799 0.8323879774839780 0.1944196861781305 T T T

0.1250000000000000 0.3300334015516881 0.1152112286609110 F F F

0.6250000000000000 0.3300334015516881 0.1152112286609110 F F F

0.1250000000000000 0.6633667348850238 0.1152112286609110 F F F

0.6250000000000000 0.6633667348850238 0.1152112286609110 F F F

0.1250000000000000 0.9967000682183524 0.1152112286609110 F F F

0.6250000000000000 0.9967000682183524 0.1152112286609110 F F F

0.3750000000000000 0.1633667348850238 0.0959918555628363 F F F

0.8750000000000000 0.1633667348850238 0.0959918555628363 F F F

0.3750000000000000 0.4967000682183524 0.0959918555628363 F F F

0.8750000000000000 0.4967000682183524 0.0959918555628363 F F F

0.3750000000000000 0.8300334015516881 0.0959918555628363 F F F

0.8750000000000000 0.8300334015516881 0.0959918555628363 F F F

0.3714610089846133 0.9962211201077140 0.2756976220127555 T T T

0.8731375341152880 0.9952851286960774 0.2756065157279013 T T T

0.3705476916193414 0.3311291617717838 0.2764676760126745 T T T

0.8914778439503990 0.3274060369481119 0.2730972325131968 T T T

0.3734490712537254 0.6673054888674007 0.2755412344587458 T T T

0.8808419943948860 0.6697801166472844 0.2735286829407613 T T T

0.1218939136777618 0.1695885280077158 0.2908750011266023 T T T

0.6164212637890424 0.1632320746539054 0.2948186575463022 T T T

0.1144354048226871 0.5072422317890315 0.2909730122493752 T T T

0.6040809596438048 0.5162494901554373 0.2903475560846706 T T T

0.1211567224933336 0.8386882556081242 0.2907307353512895 T T T

0.6199135825771460 0.8378160518865905 0.2913883022977274 T T T

0.0171000009162086 0.0927333371077097 0.0754446512056361 F F F

0.5171000009162086 0.0927333371077097 0.0754446512056361 F F F

0.0171000009162086 0.4260666704410454 0.0754446512056361 F F F

0.5171000009162086 0.4260666704410454 0.0754446512056361 F F F

0.0171000009162086 0.7594000037743740 0.0754446512056361 F F F

0.5171000009162086 0.7594000037743740 0.0754446512056361 F F F

0.4859637622911894 0.2584330450393149 0.1411267686365957 T T T

0.9814531269304598 0.2582592246846078 0.1396083987812255 T T T

0.4866986252613101 0.5898691189211365 0.1407543110040308 T T T

0.9843023580233975 0.5908907598579732 0.1395559690813145 T T T

0.4835909909597048 0.9245208687339255 0.1395418356233561 T T T

0.9825955460809265 0.9250826360015151 0.1392993489786910 T T T

0.2663079767628189 0.2325422833770454 0.3150691956838600 T T T

0.7497371687659028 0.2245310395494924 0.3276846928326237 T T T

0.2482517502693604 0.5646710594309848 0.3215513355501532 T T T

0.7371828614582651 0.5397221371402078 0.3292604184626108 T T T

0.2593191402591171 0.8996051054589560 0.3176316046464820 T T T

0.7581608046733446 0.8975587297752687 0.3179204314355789 T T T

0.2313555708651801 0.0712839045693845 0.2562017695489802 T T T

0.7314197090559863 0.0702836265970917 0.2578161792773311 T T T

0.2340307564953736 0.4061488877806213 0.2562118900412744 T T T

0.7266106031701987 0.3865395983798651 0.2704351392166051 T T T

0.2341246447193293 0.7384650558217589 0.2556675775388801 T T T

0.7344132297460154 0.7354266536715649 0.2570883704239210 T T T

0.4873147271480139 0.2350793766505770 0.3265469692918739 T T T

0.9718366903639858 0.2308637104768229 0.3173153395423357 T T T

0.4680917362058404 0.5686131543091779 0.3180641423806633 T T T

0.9645963755272011 0.5674891187818676 0.3144269153830460 T T T

0.4795689468265311 0.8972009869164186 0.3181252407973922 T T T

0.9781483985192446 0.8957366110810459 0.3173540620927291 T T T

0.0151365661905552 0.0706908665555113 0.2567019261687584 T T T

0.5148090006900532 0.0691405819275500 0.2580110420603602 T T T

0.0191021204773094 0.4064817168722737 0.2556860495150718 T T T

0.5147903411109024 0.4048847264812770 0.2604049717922196 T T T

0.0187750813655214 0.7394138118171889 0.2555108713310700 T T T

0.5182508985387499 0.7361285958086760 0.2572425455690499 T T T

0.2328999990837914 0.0927333371077097 0.0754446512056361 F F F

0.7328999990837914 0.0927333371077097 0.0754446512056361 F F F

0.2328999990837914 0.4260666704410454 0.0754446512056361 F F F

0.7328999990837914 0.4260666704410454 0.0754446512056361 F F F

0.2328999990837914 0.7594000037743740 0.0754446512056361 F F F

0.7328999990837914 0.7594000037743740 0.0754446512056361 F F F

0.2669287071149880 0.2576839866828087 0.1402591112190166 T T T

0.7627655477780094 0.2578607258982201 0.1405609586054272 T T T

0.2684133811877202 0.5904481377153518 0.1399205908651255 T T T

0.7672320907958367 0.5922815205268881 0.1385205111226192 T T T

0.2658432425551481 0.9242658481939103 0.1397511350587700 T T T

0.7647121295461515 0.9243692116252215 0.1398717793069434 T T T

0.1250000000000000 0.2554999946180203 0.0502498420463695 F F F

0.6250000000000000 0.2554999946180203 0.0502498420463695 F F F

0.1250000000000000 0.5888333279513489 0.0502498420463695 F F F

0.6250000000000000 0.5888333279513489 0.0502498420463695 F F F

0.1250000000000000 0.9221666612846846 0.0502498420463695 F F F

0.6250000000000000 0.9221666612846846 0.0502498420463695 F F F

0.1234230092719739 0.0720681432934457 0.1791990632371299 T T T

0.6232496625590142 0.0710482126564246 0.1804194828716975 T T T

0.1261713341116959 0.4054340354372611 0.1788010721789075 T T T

0.6316503255744068 0.4033656579922529 0.1859583070158679 T T T

0.1259447902380968 0.7385527853285899 0.1784359403962592 T T T

0.6260286046996061 0.7382161269046960 0.1794783682969412 T T T

0.3767665293013321 0.4205213619400027 0.1664829726110779 T T T

0.8733028526774117 0.4208160599506519 0.1669038624358684 T T T

0.3766145709640055 0.7536558485484389 0.1658065033935469 T T T

0.8755183355939009 0.7549294157290410 0.1655186640367800 T T T

0.3744452943200980 0.0877367821591420 0.1657611450295554 T T T

0.8729199804660227 0.0879757926989476 0.1656768238453867 T T T

0.3512552049794853 0.2352379382028077 0.3959978215411196 T T T

0.8951112899161036 0.2267109930055014 0.3983747867253326 T T T

0.3672731618459997 0.5743694357201059 0.3961252264407546 T T T

0.8734929998200088 0.5741596703972842 0.3951102864302780 T T T

0.3683142391353155 0.9072294099922162 0.3941607840093401 T T T

0.8690213482587036 0.9020467233612667 0.3939509676639388 T T T

0.3708033887139233 0.0720450431738513 0.3451062430050273 T T T

0.8705654062843889 0.0686895060560493 0.3466228340998282 T T T

0.3660012026298167 0.4073159206080231 0.3474696988636722 T T T

0.8797919299361207 0.4033740453278438 0.3458433331237612 T T T

0.3667492383986994 0.7392089045487941 0.3456352270566411 T T T

0.8647206267301750 0.7356247640989394 0.3440300459129482 T T T

0.3745906211402709 0.2574163587952910 0.2168877990796020 T T T

0.8729291096187168 0.2567967171337421 0.2159044445537926 T T T

0.3765009335719998 0.5904795287673301 0.2165797616733077 T T T

0.8734053401994739 0.5910134508190128 0.2153341806578941 T T T

0.3751043845336456 0.9240270724440203 0.2158068794017703 T T T

0.8745693595426918 0.9241084867159440 0.2156880982338675 T T T

0.1240560095899233 0.9085255916662378 0.2290923065737349 T T T

0.6240234986129969 0.9074743730726229 0.2298836360777632 T T T

0.1223973401532546 0.2427738211707139 0.2299727283798081 T T T

0.6199001666031005 0.2382482260781870 0.2333174487160304 T T T

0.1238896716866005 0.5759520638359198 0.2293135995914516 T T T

0.6264558796643899 0.5747004008813221 0.2295076393116970 T T T

0.1250000000000000 0.0936333333333366 0.0000000000000000 F F F

0.6250000000000000 0.0936333333333366 0.0000000000000000 F F F

0.1250000000000000 0.4269666666666652 0.0000000000000000 F F F

0.6250000000000000 0.4269666666666652 0.0000000000000000 F F F

0.1250000000000000 0.7603000000000009 0.0000000000000000 F F F

0.6250000000000000 0.7603000000000009 0.0000000000000000 F F F

0.6292772712646021 0.3637309813635564 0.3935883735879650 T T T

0.7559140125842664 0.3319044121883142 0.4486210018818084 T T T

0.1250000000000000 0.1423333253652075 0.0512282806593802 F F F

0.6250000000000000 0.1423333253652075 0.0512282806593802 F F F

0.1250000000000000 0.4756666586985361 0.0512282806593802 F F F

0.6250000000000000 0.4756666586985361 0.0512282806593802 F F F

0.1250000000000000 0.8089999920318718 0.0512282806593802 F F F

0.6250000000000000 0.8089999920318718 0.0512282806593802 F F F

0.3756807499853376 0.3069825508987450 0.1643472643355342 T T T

0.8723866380712260 0.3072446664628759 0.1634821035145392 T T T

0.3770562538743403 0.6402134828064097 0.1639747816471647 T T T

0.8758735981511541 0.6418090002460954 0.1629648640017956 T T T

0.3747556122080147 0.9742044165338944 0.1634592521823394 T T T

0.8737496986523344 0.9744188321831387 0.1633611666840627 T T T

0.3699089989956607 0.1860031076807943 0.3456238247865127 T T T

0.8680412671118064 0.1818692279956743 0.3459682316641641 T T T

0.3608136605834958 0.5217612199533622 0.3460075406359955 T T T

0.8668567983057546 0.5181410662049054 0.3464031601003152 T T T

0.3683867511125150 0.8536944173162582 0.3444930642877504 T T T

0.8676065862860836 0.8503027479333014 0.3438032540424597 T T T

0.1233298539003547 0.0224357996440572 0.2302872412168172 T T T

0.6235931194552776 0.0215769966543464 0.2314891133460664 T T T

0.1277089524925490 0.3567755730350418 0.2298849050046506 T T T

0.6231261134747642 0.3517045365456792 0.2362593899947113 T T T

0.1265335233508491 0.6900113115468784 0.2295771848155245 T T T

0.6262714059470992 0.6896332521522759 0.2304881142620068 T T T

0.4079364047209112 0.3837068326452830 0.4749482101971744 T T T

0.4462798022031023 0.4738211857855630 0.4433590562323391 T T T

0.5794110494179658 0.3477265122689471 0.5004025650756513 T T T

0.6119448340965440 0.4636975788778999 0.4852158987386790 T T T

0.8357160782762040 0.2539337763188152 0.4182371629552373 T T T

0.6258942355443311 0.6422828712758963 0.5132461993383373 T T T

0.6409177392402929 0.6236623815218982 0.5364310953984037 T T T

0.5797772359139834 0.3970461543642566 0.4717048428683753 T T T

0.6638681010208257 0.3613633603160359 0.4368282973097921 T T T

0.4666907192739984 0.4055903113868511 0.4518404140465364 T T T

**Glycine formation, Mechanism 2: Reactant**

Mg O Si H C N

1.00000000000000

12.0060000000000002 0.0000000000000000 0.0000000000000000

0.0000000000000000 14.3070000000000004 0.0000000000000000

0.0000000000000000 0.0000000000000000 29.3124000000000002

Mg O Si H C N

48 98 24 7 2 1

Selective dynamics

Direct

0.0022707696690500 0.9934364907589190 0.3736204216393041 T T T

0.5017803885076098 0.9937897841238449 0.3736822659113798 T T T

0.0005833686286506 0.3282528010616058 0.3734570168992281 T T T

0.4881284629931594 0.3313699715889505 0.3850046926457816 T T T

0.0005304559736654 0.6607649473836787 0.3740095324683544 T T T

0.5020641637791704 0.6620906760585141 0.3727353163681421 T T T

0.9997467421193190 0.1650535465453218 0.1940141965640920 T T T

0.4997049199990237 0.1651857769319993 0.1940431173535825 T T T

-0.0003118592744136 0.4984409217045720 0.1939676808604704 T T T

0.4994416997998106 0.4983956977844908 0.1938270695525234 T T T

0.9992591157629708 0.8317006945897641 0.1940357186109873 T T T

0.4992618595979260 0.8320156983940485 0.1940654807493672 T T T

0.2475006036372427 0.9944790942469234 0.3737936313084021 T T T

0.7474089970207802 0.9939642595589140 0.3738840946597380 T T T

0.2415334748913321 0.3256129029886842 0.3716691696478804 T T T

0.7483037089415492 0.3250658261572568 0.3717566641254744 T T T

0.2460399166630393 0.6616988665021312 0.3743813369979871 T T T

0.7457609842472859 0.6617258650187794 0.3735332567482209 T T T

0.2500150867492066 0.1651933646132490 0.1941406856435338 T T T

0.7497363434117310 0.1650283933590288 0.1942833829811307 T T T

0.2498423911015429 0.4985059837477359 0.1941774017277920 T T T

0.7497126679823189 0.4983930347926926 0.1939731671559539 T T T

0.2494887828457457 0.8318172978819035 0.1939694712701337 T T T

0.7494380201389039 0.8319466669021164 0.1939966236632612 T T T

0.1250000000000000 0.3300334015516881 0.1152112286609110 F F F

0.6250000000000000 0.3300334015516881 0.1152112286609110 F F F

0.1250000000000000 0.6633667348850238 0.1152112286609110 F F F

0.6250000000000000 0.6633667348850238 0.1152112286609110 F F F

0.1250000000000000 0.9967000682183524 0.1152112286609110 F F F

0.6250000000000000 0.9967000682183524 0.1152112286609110 F F F

0.3750000000000000 0.1633667348850238 0.0959918555628363 F F F

0.8750000000000000 0.1633667348850238 0.0959918555628363 F F F

0.3750000000000000 0.4967000682183524 0.0959918555628363 F F F

0.8750000000000000 0.4967000682183524 0.0959918555628363 F F F

0.3750000000000000 0.8300334015516881 0.0959918555628363 F F F

0.8750000000000000 0.8300334015516881 0.0959918555628363 F F F

0.3742813927137065 0.9987013602709152 0.2748091339435654 T T T

0.8746033479911292 0.9983439285121758 0.2746468092361697 T T T

0.3752449568808792 0.3316331935143448 0.2754352723058998 T T T

0.8750593027396072 0.3317583410290149 0.2742213710075206 T T T

0.3739590207607615 0.6652602608948190 0.2745975233477338 T T T

0.8744816836353982 0.6651965472221185 0.2746029938995259 T T T

0.1259560697282747 0.1723552010017593 0.2899294572503347 T T T

0.6232084471249361 0.1729425089634597 0.2908092605107639 T T T

0.1228518928991952 0.5060009362654904 0.2903445142319745 T T T

0.6227776363120378 0.5067024020163126 0.2894767690227698 T T T

0.1242693523235436 0.8391477030639453 0.2900502203578763 T T T

0.6243448867421911 0.8390247667309518 0.2901038562862593 T T T

0.0171000009162086 0.0927333371077097 0.0754446512056361 F F F

0.5171000009162086 0.0927333371077097 0.0754446512056361 F F F

0.0171000009162086 0.4260666704410454 0.0754446512056361 F F F

0.5171000009162086 0.4260666704410454 0.0754446512056361 F F F

0.0171000009162086 0.7594000037743740 0.0754446512056361 F F F

0.5171000009162086 0.7594000037743740 0.0754446512056361 F F F

0.4837317257270831 0.2575464971123672 0.1392400167522884 T T T

0.9835666926800746 0.2574995032041516 0.1391129956607960 T T T

0.4833461293190273 0.5909737564025158 0.1391475046938911 T T T

0.9834286715830944 0.5908894242501471 0.1391738646237289 T T T

0.4835022402153347 0.9242992457194736 0.1392244519739839 T T T

0.9834456032723284 0.9242736056427419 0.1392288371191534 T T T

0.2679781986444862 0.2330757177734357 0.3163216124128568 T T T

0.7642250338324227 0.2324011472434789 0.3176188684429965 T T T

0.2616861213192891 0.5662861536976730 0.3188349879295505 T T T

0.7614820158132068 0.5660524831286736 0.3178178881108811 T T T

0.2635747077171335 0.8996527136257225 0.3177617922186459 T T T

0.7632633133053145 0.8994871055720528 0.3179187621355452 T T T

0.2326178077404682 0.0713798021117939 0.2561818081381437 T T T

0.7326246673403445 0.0715955565205578 0.2565293200317041 T T T

0.2328777829557678 0.4052443774307135 0.2565624800137072 T T T

0.7324030025118523 0.4049496589540780 0.2564978308328544 T T T

0.2323502439288961 0.7382107815142575 0.2562428854862668 T T T

0.7324853762458999 0.7382478307950674 0.2560644997877725 T T T

0.4897689257455662 0.2344210382380645 0.3221734543090273 T T T

0.9854871509396307 0.2325145025696513 0.3174747266915376 T T T

0.4828853183881276 0.5656737314033862 0.3177084897216863 T T T

0.9829917887579908 0.5664619252041313 0.3177106714603618 T T T

0.4850067852256443 0.8990167898799886 0.3178633002793259 T T T

0.9847330135743759 0.8991546504596797 0.3176105124025126 T T T

0.0165858517430021 0.0714446321191353 0.2561129496905766 T T T

0.5164417740585177 0.0721003709805611 0.2567013529835382 T T T

0.0166464313428693 0.4050436095962692 0.2562591140163478 T T T

0.5163959409323781 0.4051036164515249 0.2564231338355335 T T T

0.0162917193424046 0.7383249156992500 0.2561549476457951 T T T

0.5163822249215799 0.7380405268302309 0.2563021016280932 T T T

0.2328999990837914 0.0927333371077097 0.0754446512056361 F F F

0.7328999990837914 0.0927333371077097 0.0754446512056361 F F F

0.2328999990837914 0.4260666704410454 0.0754446512056361 F F F

0.7328999990837914 0.4260666704410454 0.0754446512056361 F F F

0.2328999990837914 0.7594000037743740 0.0754446512056361 F F F

0.7328999990837914 0.7594000037743740 0.0754446512056361 F F F

0.2661038239353977 0.2575555148192907 0.1393401654182712 T T T

0.7657884103682739 0.2574248722776738 0.1392408392275061 T T T

0.2657354123131846 0.5908469205578486 0.1393079429486786 T T T

0.7657710652255716 0.5908375770713028 0.1392419962090992 T T T

0.2659219539482346 0.9242489656350596 0.1392164220490874 T T T

0.7658180557832005 0.9242526842824403 0.1392090344528361 T T T

0.1250000000000000 0.2554999946180203 0.0502498420463695 F F F

0.6250000000000000 0.2554999946180203 0.0502498420463695 F F F

0.1250000000000000 0.5888333279513489 0.0502498420463695 F F F

0.6250000000000000 0.5888333279513489 0.0502498420463695 F F F

0.1250000000000000 0.9221666612846846 0.0502498420463695 F F F

0.6250000000000000 0.9221666612846846 0.0502498420463695 F F F

0.1248757361223173 0.0720026044800232 0.1788395634464494 T T T

0.6245215315256664 0.0719760415072663 0.1791885239558343 T T T

0.1250032462691411 0.4052829905370461 0.1790413274910988 T T T

0.6246678485273344 0.4050252374398705 0.1790773981967921 T T T

0.1244696600240697 0.7384892154142628 0.1788413571315413 T T T

0.6242368039029994 0.7389021538385858 0.1787886268731158 T T T

0.3747508744579895 0.4210619066132997 0.1650465837377559 T T T

0.8747389153686895 0.4209188852931947 0.1650832473346710 T T T

0.3745741932499719 0.7543526040399291 0.1650981788585335 T T T

0.8744966639042041 0.7542878268827599 0.1650431007047466 T T T

0.3747895243269969 0.0877182375185961 0.1652070863856977 T T T

0.8745915362436837 0.0876698071391278 0.1652111756063295 T T T

0.3647556500317992 0.2383190001719499 0.3951691499807718 T T T

0.8742722909058921 0.2409824705070235 0.3934024412074156 T T T

0.3752594321346358 0.5741042965008192 0.3944414879930385 T T T

0.8722991123686188 0.5744454723254749 0.3937657031108181 T T T

0.3742834468447766 0.9077358220185560 0.3937720921796851 T T T

0.8745809522865859 0.9076515085240465 0.3937258132444433 T T T

0.3751807891308037 0.0733224078858463 0.3452336678615572 T T T

0.8749403772580481 0.0726648663234563 0.3451200575755627 T T T

0.3725754922607477 0.4064882502150817 0.3465295730618327 T T T

0.8727046432660032 0.4067112842137212 0.3449738608039482 T T T

0.3733482626713819 0.7397451845996686 0.3452814738584011 T T T

0.8736982722171128 0.7398116005762357 0.3451552521147546 T T T

0.3752501355496952 0.2579259180270398 0.2156803526039293 T T T

0.8747576037533673 0.2575654219185424 0.2153800414932046 T T T

0.3746502685006667 0.5909887573184792 0.2155061271738639 T T T

0.8745787740427380 0.5908387933794184 0.2154522495026058 T T T

0.3745500709702632 0.9244227122998719 0.2155315510181091 T T T

0.8744370599846986 0.9242310247685012 0.2154807338095173 T T T

0.1245377383425376 0.9087947334802235 0.2288802435170074 T T T

0.6242092856776307 0.9091455223789480 0.2291380699997335 T T T

0.1248975021034386 0.2423756973602461 0.2290362516944438 T T T

0.6242572250526890 0.2422353681244747 0.2294456884618974 T T T

0.1243699321711806 0.5755668830758685 0.2290959469142758 T T T

0.6242093484595371 0.5757378379079858 0.2286149784881150 T T T

0.1250000000000000 0.0936333333333366 0.0000000000000000 F F F

0.6250000000000000 0.0936333333333366 0.0000000000000000 F F F

0.1250000000000000 0.4269666666666652 0.0000000000000000 F F F

0.6250000000000000 0.4269666666666652 0.0000000000000000 F F F

0.1250000000000000 0.7603000000000009 0.0000000000000000 F F F

0.6250000000000000 0.7603000000000009 0.0000000000000000 F F F

0.3849376978548226 0.3473416251567946 0.5446592583273873 T T T

0.3773023515237934 0.3333191260420880 0.6220890661236645 T T T

0.1250000000000000 0.1423333253652075 0.0512282806593802 F F F

0.6250000000000000 0.1423333253652075 0.0512282806593802 F F F

0.1250000000000000 0.4756666586985361 0.0512282806593802 F F F

0.6250000000000000 0.4756666586985361 0.0512282806593802 F F F

0.1250000000000000 0.8089999920318718 0.0512282806593802 F F F

0.6250000000000000 0.8089999920318718 0.0512282806593802 F F F

0.3749642892466246 0.3075068549544647 0.1631959509810064 T T T

0.8747166069633329 0.3073733770158331 0.1629769449747309 T T T

0.3745756318281752 0.6408540196868848 0.1630745408692152 T T T

0.8746219565800558 0.6407829432553143 0.1630660753770948 T T T

0.3746838418623745 0.9742015409172476 0.1631205238963041 T T T

0.8746105502789994 0.9741592170268383 0.1631037697201909 T T T

0.3758966297324470 0.1878234425281165 0.3448306274323190 T T T

0.8754196389024529 0.1867182236505970 0.3436484253782842 T T T

0.3726561231058022 0.5205976936425561 0.3442268191102643 T T T

0.8723704522567655 0.5211805973935414 0.3438812150784598 T T T

0.3739993918427682 0.8541611667747899 0.3439744282292924 T T T

0.8741168907276016 0.8540884199721078 0.3439092280895135 T T T

0.1245894488624213 0.0228130590089052 0.2299911862900063 T T T

0.6244626623984194 0.0232130449698640 0.2304517818695110 T T T

0.1251211710875613 0.3564346123289237 0.2302718156288838 T T T

0.6244248429952115 0.3562476168485544 0.2303156045787967 T T T

0.1243413082148007 0.6896131819412897 0.2300572678351627 T T T

0.6243742294948338 0.6897949044699370 0.2299275842099351 T T T

0.4241070289853075 0.3758515544998700 0.4713651507337751 T T T

0.4326216013539479 0.4708473404399109 0.4389867298944863 T T T

0.6322726689282382 0.4795194422833201 0.4525332095440919 T T T

0.6208028178415177 0.3767462705955785 0.4863893859806908 T T T

0.2990687485623198 0.3171908464742660 0.6161245222515045 T T T

0.5550465009649008 0.4822334177393440 0.5046170343196363 T T T

0.5163292680064886 0.3690370747121927 0.5888191145662321 T T T

0.5761751638946523 0.4393041442567844 0.4746996405016952 T T T

0.4282422703692436 0.3507904769909712 0.5823238346282982 T T T

0.4754023774643212 0.4109173833110448 0.4487528000910369 T T T

**Glycine formation, Mechanism 2: Product**

Mg O Si H C N

1.00000000000000

12.0060000000000002 0.0000000000000000 0.0000000000000000

0.0000000000000000 14.3070000000000004 0.0000000000000000

0.0000000000000000 0.0000000000000000 29.3124000000000002

Mg O Si H C N

48 98 24 7 2 1

Selective dynamics

Direct

0.0036327657089059 0.9935682593121948 0.3737245575516144 T T T

0.5032100571436471 0.9938417682099560 0.3736296206794125 T T T

0.0017693226124218 0.3283346657505396 0.3734110555095544 T T T

0.4901434451050174 0.3309622311822411 0.3835211778660176 T T T

0.0020816445209822 0.6605805841676052 0.3741228502392522 T T T

0.5035600000162481 0.6618806122503975 0.3725350023965892 T T T

0.0001735798646605 0.1650529653300959 0.1939601791023602 T T T

0.5002372544350019 0.1651799792807504 0.1940572178586791 T T T

0.0002919457819761 0.4983785920190223 0.1939590428750071 T T T

0.5001495447131113 0.4983457285777765 0.1938454699809726 T T T

0.0002458142943084 0.8316665799585014 0.1939788844169529 T T T

0.5003205864105602 0.8320104816353816 0.1940370270471752 T T T

0.2490131445793922 0.9942506676446725 0.3736528415762939 T T T

0.7490424094093482 0.9939299155320116 0.3737388347027055 T T T

0.2428798511764103 0.3255717662189425 0.3709625790397781 T T T

0.7491674544520148 0.3252199805878493 0.3722737531541436 T T T

0.2475611278772296 0.6615439593971235 0.3743041117677530 T T T

0.7473907682347216 0.6614547615624342 0.3738099803136458 T T T

0.2505075226988458 0.1650927616165101 0.1940696666353781 T T T

0.7502365769771490 0.1649823613023311 0.1942328902730422 T T T

0.2504616124568312 0.4984669893007708 0.1940505171479458 T T T

0.7503992168646105 0.4983527950869387 0.1938779485427728 T T T

0.2505289731614553 0.8317773927967341 0.1939616398711529 T T T

0.7504731657205479 0.8318915729624647 0.1940106169776088 T T T

0.1250000000000000 0.3300334015516881 0.1152112286609110 F F F

0.6250000000000000 0.3300334015516881 0.1152112286609110 F F F

0.1250000000000000 0.6633667348850238 0.1152112286609110 F F F

0.6250000000000000 0.6633667348850238 0.1152112286609110 F F F

0.1250000000000000 0.9967000682183524 0.1152112286609110 F F F

0.6250000000000000 0.9967000682183524 0.1152112286609110 F F F

0.3750000000000000 0.1633667348850238 0.0959918555628363 F F F

0.8750000000000000 0.1633667348850238 0.0959918555628363 F F F

0.3750000000000000 0.4967000682183524 0.0959918555628363 F F F

0.8750000000000000 0.4967000682183524 0.0959918555628363 F F F

0.3750000000000000 0.8300334015516881 0.0959918555628363 F F F

0.8750000000000000 0.8300334015516881 0.0959918555628363 F F F

0.3750567903019051 0.9986329618831060 0.2747431100517980 T T T

0.8754198614507497 0.9982852944730629 0.2746392008054409 T T T

0.3761054472183545 0.3317177332557067 0.2751804342143048 T T T

0.8757847583992426 0.3317346881905376 0.2743120917710595 T T T

0.3751689878143485 0.6653203179780213 0.2744838761609158 T T T

0.8756754125432190 0.6650116800050013 0.2746266551002436 T T T

0.1265516891353673 0.1722417394580840 0.2897468258886830 T T T

0.6245385304901981 0.1728813903106819 0.2907704664539602 T T T

0.1241929736656159 0.5059904498059835 0.2902306207375717 T T T

0.6245104075478216 0.5066557037217434 0.2894212156281228 T T T

0.1261058428989126 0.8390699301598449 0.2899933721619116 T T T

0.6263646249345294 0.8389471052806490 0.2900799079435134 T T T

0.0171000009162086 0.0927333371077097 0.0754446512056361 F F F

0.5171000009162086 0.0927333371077097 0.0754446512056361 F F F

0.0171000009162086 0.4260666704410454 0.0754446512056361 F F F

0.5171000009162086 0.4260666704410454 0.0754446512056361 F F F

0.0171000009162086 0.7594000037743740 0.0754446512056361 F F F

0.5171000009162086 0.7594000037743740 0.0754446512056361 F F F

0.4841161926745840 0.2575016876478192 0.1392085555837319 T T T

0.9838144765330294 0.2574411969095482 0.1390840974968262 T T T

0.4840442855591234 0.5908809310448296 0.1391864041452525 T T T

0.9840881494665703 0.5908283720356748 0.1392110450833311 T T T

0.4840886640541796 0.9242335398424487 0.1392039413670833 T T T

0.9839698789165507 0.9241817814610892 0.1391858952291995 T T T

0.2687470269913094 0.2327047572584323 0.3158822591998522 T T T

0.7653135233299735 0.2323815384353155 0.3177092600846425 T T T

0.2632341809430437 0.5666621511490088 0.3185966926872283 T T T

0.7632192224287105 0.5662682385304660 0.3177424465986387 T T T

0.2658781083745481 0.8995767792104541 0.3174510623742576 T T T

0.7655552924285145 0.8994336533261863 0.3177000510379887 T T T

0.2332154307698311 0.0712867591711259 0.2560333701488218 T T T

0.7332837720863031 0.0715514293317849 0.2565134150984926 T T T

0.2333529812153253 0.4051391743906118 0.2564655406252302 T T T

0.7330911904310035 0.4049148003036626 0.2564224123090250 T T T

0.2334365405485057 0.7382219173363369 0.2561498654661857 T T T

0.7336545066292255 0.7382645595390431 0.2560126452563899 T T T

0.4904026301108917 0.2342083746578513 0.3216881129633047 T T T

0.9866144668564476 0.2326148582000392 0.3174081431103465 T T T

0.4844540173857992 0.5657730441190514 0.3174757096890500 T T T

0.9846413733471170 0.5664101977488567 0.3177307989396975 T T T

0.4873159312546235 0.8987445018214282 0.3180291623237659 T T T

0.9869993977675761 0.8989966087923876 0.3178289200935006 T T T

0.0171578638947897 0.0713716291002272 0.2560484390379384 T T T

0.5171070686614885 0.0719495921205892 0.2566404835474580 T T T

0.0171786773134476 0.4051092722958170 0.2561100882917336 T T T

0.5170958608205013 0.4052495309795641 0.2562725420582204 T T T

0.0174159482264023 0.7382151097267833 0.2560938030157192 T T T

0.5176105196817636 0.7379691918330938 0.2563060977785586 T T T

0.2328999990837914 0.0927333371077097 0.0754446512056361 F F F

0.7328999990837914 0.0927333371077097 0.0754446512056361 F F F

0.2328999990837914 0.4260666704410454 0.0754446512056361 F F F

0.7328999990837914 0.4260666704410454 0.0754446512056361 F F F

0.2328999990837914 0.7594000037743740 0.0754446512056361 F F F

0.7328999990837914 0.7594000037743740 0.0754446512056361 F F F

0.2665358467045729 0.2574870789735102 0.1392409863144862 T T T

0.7660869578855304 0.2573762923731217 0.1392003618126844 T T T

0.2664214892684216 0.5909115132252619 0.1391842566550376 T T T

0.7664366185790656 0.5909006249167341 0.1391276443854834 T T T

0.2665177812346697 0.9242728501077913 0.1392061232750056 T T T

0.7663349698874250 0.9242338997660443 0.1391876055198903 T T T

0.1250000000000000 0.2554999946180203 0.0502498420463695 F F F

0.6250000000000000 0.2554999946180203 0.0502498420463695 F F F

0.1250000000000000 0.5888333279513489 0.0502498420463695 F F F

0.6250000000000000 0.5888333279513489 0.0502498420463695 F F F

0.1250000000000000 0.9221666612846846 0.0502498420463695 F F F

0.6250000000000000 0.9221666612846846 0.0502498420463695 F F F

0.1252425704239346 0.0719521715661671 0.1787528239542124 T T T

0.6250477865280534 0.0719476339904089 0.1791618790016259 T T T

0.1255373696362039 0.4052273949739494 0.1789274133367079 T T T

0.6252878284116278 0.4049838792929837 0.1789961982897920 T T T

0.1254834700372046 0.7384836844953393 0.1787805183455851 T T T

0.6252155360814973 0.7388913715679962 0.1787698260893410 T T T

0.3752517496570385 0.4210346620579101 0.1650032917456698 T T T

0.8751922797787741 0.4208984125577556 0.1650037445248775 T T T

0.3753760514591386 0.7543263321713036 0.1650846600371400 T T T

0.8752559205858155 0.7542680977336094 0.1650284345383776 T T T

0.3753901367098597 0.0876776848999600 0.1651884097957076 T T T

0.8750954380711344 0.0876253898794027 0.1651621610415545 T T T

0.3657497018894595 0.2381855275343373 0.3945859584014419 T T T

0.8759370694690068 0.2410228777599454 0.3934219018962214 T T T

0.3769655496976733 0.5736390966454501 0.3941912824918777 T T T

0.8740413798135082 0.5742814494895108 0.3937519995714535 T T T

0.3757426038133170 0.9077647597979459 0.3936900427920727 T T T

0.8760749310089319 0.9077661457131421 0.3937283675739569 T T T

0.3763826293097490 0.0729533291478788 0.3448993859004560 T T T

0.8763926466748244 0.0727508344703735 0.3451045530349915 T T T

0.3735853837123967 0.4064361283908947 0.3456958580884991 T T T

0.8738507095708098 0.4066633695137396 0.3449188469889949 T T T

0.3749578871004695 0.7396797600624757 0.3452949716010043 T T T

0.8752792034951609 0.7397693476418159 0.3453374692390585 T T T

0.3756388831400015 0.2578682012857786 0.2155784340712084 T T T

0.8751247710170708 0.2575691571588525 0.2153560754307683 T T T

0.3752736087158718 0.5908997907308885 0.2154461717045613 T T T

0.8752047457772001 0.5907596457889747 0.2154110996525923 T T T

0.3754572745417867 0.9243491931915552 0.2154980932866230 T T T

0.8752570783923160 0.9242097489944319 0.2154470878140014 T T T

0.1253715686244836 0.9087190147370755 0.2288027415614367 T T T

0.6251993293332989 0.9090902474941153 0.2291144604821539 T T T

0.1252994344730885 0.2423468499560619 0.2288754775503435 T T T

0.6248445677140669 0.2422189708143560 0.2294063594481485 T T T

0.1253800034431781 0.5755640441548914 0.2289878384060396 T T T

0.6253375468171692 0.5757457379109515 0.2285553990950908 T T T

0.1250000000000000 0.0936333333333366 0.0000000000000000 F F F

0.6250000000000000 0.0936333333333366 0.0000000000000000 F F F

0.1250000000000000 0.4269666666666652 0.0000000000000000 F F F

0.6250000000000000 0.4269666666666652 0.0000000000000000 F F F

0.1250000000000000 0.7603000000000009 0.0000000000000000 F F F

0.6250000000000000 0.7603000000000009 0.0000000000000000 F F F

0.3982246162869447 0.3642442725305619 0.5354821002951792 T T T

0.5680027020451088 0.4022000185765051 0.5648664196354326 T T T

0.1250000000000000 0.1423333253652075 0.0512282806593802 F F F

0.6250000000000000 0.1423333253652075 0.0512282806593802 F F F

0.1250000000000000 0.4756666586985361 0.0512282806593802 F F F

0.6250000000000000 0.4756666586985361 0.0512282806593802 F F F

0.1250000000000000 0.8089999920318718 0.0512282806593802 F F F

0.6250000000000000 0.8089999920318718 0.0512282806593802 F F F

0.3753491014216708 0.3074741441458554 0.1631177908328613 T T T

0.8750054550794008 0.3073588236946568 0.1629526767026434 T T T

0.3752310640334383 0.6408148426237180 0.1630402474639740 T T T

0.8752446267936403 0.6407504998908263 0.1630469860467167 T T T

0.3753291499925351 0.9741671749172762 0.1631030091561871 T T T

0.8751745517214548 0.9741127392495893 0.1630766598536678 T T T

0.3766561465229964 0.1874435562606312 0.3443570993521905 T T T

0.8766121327402585 0.1868721083334798 0.3436690345935628 T T T

0.3741508445639186 0.5205765892652092 0.3438298564267165 T T T

0.8739241997779919 0.5211028876391770 0.3438599902424259 T T T

0.3759723922360595 0.8540743382816377 0.3439233498419644 T T T

0.8761036601975342 0.8540202235747021 0.3439514689018675 T T T

0.1251273868438549 0.0227360422140064 0.2298959235195100 T T T

0.6251237361070254 0.0231483253955962 0.2304203808081411 T T T

0.1255657219224437 0.3563920712495401 0.2301639027766558 T T T

0.6250931275254676 0.3562385840057491 0.2302496132047828 T T T

0.1253700590388692 0.6895810685308672 0.2299877018771808 T T T

0.6254970782265754 0.6897606430516890 0.2299035662038219 T T T

0.3937219827043555 0.3717156321432608 0.4626868121547829 T T T

0.4323372494840454 0.4716679095276528 0.4389906656271901 T T T

0.5846384314506594 0.4819715564868230 0.4851668325863316 T T T

0.6139406658072510 0.3621781403826066 0.4787554009403799 T T T

0.5310837349890550 0.3853772746789878 0.5936229513157329 T T T

0.8366257815600667 0.4171393392279295 0.5115546826697301 T T T

0.7995520063387118 0.4088845633328896 0.5318165907036643 T T T

0.5456431833104404 0.4125300693325180 0.4844201006044133 T T T

0.4941281935050805 0.3907759429423222 0.5306542292334553 T T T

0.4611466005652724 0.4057069656226009 0.4479390100546001 T T T

**Glycine formation, Mechanism 3: Reactant**

O H C N

1.00000000000000

20.0000000000000000 0.0000000000000000 0.0000000000000000

0.0000000000000000 20.0000000000000000 0.0000000000000000

0.0000000000000000 0.0000000000000000 20.0000000000000000

O H C N

2 7 2 1

Direct

0.3313107696245497 0.2404310650456015 0.2270227814530930

0.2629299492479164 0.3261870570002244 0.2561316795747594

0.3055603067873963 0.3498229423472174 0.2507233484820801

0.2183364196366876 0.1778458377661559 0.2112818029124780

0.2232977349342826 0.1899324198238027 0.2985554709548260

0.1120516071501314 0.2196531681039947 0.2528912359635460

0.1479932711610114 0.2899480165830311 0.2785281684691015

0.3354987188945318 0.0681222006417973 0.2136709151263292

0.3408264520964080 0.1053137599974708 0.2158374006943585

0.2757614341717022 0.2606740124671348 0.2420099514176692

0.2149470943297380 0.2163391550015463 0.2503551644904902

0.1512732799656474 0.2515870047220166 0.2447186694612603

**Glycine formation, Mechanism 3: Reactant**

O H C N

1.00000000000000

20.0000000000000000 0.0000000000000000 0.0000000000000000

0.0000000000000000 20.0000000000000000 0.0000000000000000

0.0000000000000000 0.0000000000000000 20.0000000000000000

O H C N

2 7 2 1

Direct

0.3939866074802841 0.2814605362277421 0.2779281132214703

0.3313979592568854 0.3686875519634369 0.2390078429968003

0.3749017717507128 0.3913742101550919 0.2414084063029348

0.1517844828602121 0.1480990238873975 0.1813337094269764

0.1589336907050313 0.1225751445506153 0.2664688356973659

0.0489391277265827 0.1771490304938738 0.2390604390731993

0.0899470376708331 0.2242145879478699 0.2928958361068109

0.1958565105687223 0.1987321649985300 0.2386801841241849

0.2929619371388557 0.2776478825248736 0.2580825120903541

0.3410253948962795 0.3049239787653168 0.2600041421189841

0.1517832721951831 0.1662612717432109 0.2332199485369577

0.0911076337504174 0.2057058397420398 0.2450855608039552
